# Targeting deubiquitinating enzymes and ubiquitin pathway modulators to enhance host defense against bacterial infections

**DOI:** 10.1128/mbio.00312-25

**Published:** 2025-09-03

**Authors:** John Santelices, Alexander Schultz, Alyssa Walker, Nicole Adams, Deyaneira Tirado, Hailey Barker, Aria Eshraghi, Daniel M. Czyż, Mariola J. Ferraro

**Affiliations:** 1 Microbiology and Cell Science Department, Institute of Food and Agricultural Sciences, University of Florida 209721, Gainesville, Florida, USA; 2 Department of Infectious Diseases & Immunology, College of Veterinary Medicine, University of Florida 828883, Gainesville, Florida, USA; University of Georgia Center for Food Safety, Griffin, Georgia, USA

**Keywords:** deubiquitinating enzymes (DUBs), ubiquitin-proteasome system (UPS), host-targeted therapies (HTTs), bacterial infection, *Salmonella*, USP25 inhibitor AZ-1, macrophage response, antimicrobial resistance (AMR), ESKAPE pathogens, immune modulation

## Abstract

The rise of antibiotic-resistant bacterial pathogens poses a critical global health challenge, necessitating innovative therapeutic strategies. This study explores host-targeted therapies by focusing on deubiquitinating enzymes (DUBs), key regulators of the ubiquitin-proteasome system (UPS) that mediate host-pathogen interactions. Using *Salmonella*-infected macrophages, we screened a UPS-targeted compound library and identified several compounds that enhanced bacterial clearance without affecting host cell viability. Among these, the dual USP25/USP28 inhibitor AZ-1 emerged as one of the top candidates. Transcriptomic profiling revealed infection-induced upregulation of DUBs, particularly USP25, USP46, and OTUD7B. USP25 knockdown significantly reduced intracellular *Salmonella*, confirming its role as a critical host factor. AZ-1 also exhibited broad-spectrum intracellular activity against multidrug-resistant *Pseudomonas aeruginosa*, *Klebsiella pneumoniae*, and *Acinetobacter baumannii*. *In vivo*, AZ-1 reduced fecal bacterial loads, clinical scores, and infection-induced weight loss, though it did not extend survival. AZ-1 had no direct antibacterial activity in axenic culture, indicating a host-targeting mechanism. Transcriptomic and signaling analyses revealed AZ-1 suppressed key immune pathways, including nuclear factor-kappa B (NF-κB) signaling. These findings establish DUBs as promising targets for host-directed therapies and support further development of UPS-targeted agents to combat antimicrobial resistance.

**IMPORTANCE:**

Antibiotic-resistant infections, particularly those caused by intracellular pathogens, represent an urgent public health threat due to their ability to evade immune responses and resist conventional antibiotics. This study identifies the ubiquitin-proteasome system, specifically deubiquitinating enzymes, as viable targets for host-directed therapy. We demonstrate that the USP25/USP28 inhibitor AZ-1 enhances intracellular bacterial clearance without compromising host cell viability and is effective against several multidrug-resistant gram-negative pathogens. Knockdown of USP25 alone also reduced intracellular *Salmonella*, stressing out its proposed role in bacterial persistence. AZ-1 improved early infection outcomes *in vivo* but was insufficient as monotherapy. These findings support a novel therapeutic approach that targets host pathways to enhance bacterial clearance, offering a promising adjunct to traditional antibiotics in the fight against antimicrobial resistance.

## INTRODUCTION

Infectious diseases continue to pose a significant global health threat, with bacterial pathogens constantly evolving new strategies to evade host immune defenses. Among these pathogens, *Salmonella* is particularly concerning due to its ability to cause severe, often life-threatening infections. This issue is compounded by the increasing prevalence of antimicrobial resistance (AMR), which renders conventional treatment ineffective (1, 2). The rising AMR in gram-negative bacteria like *Salmonella* fuels the urgent need for novel therapeutic approaches that extend beyond traditional antibiotics. One promising approach to combat resistant pathogens involves targeting components of the host’s cellular machinery, such as the ubiquitin-proteasome system (UPS). The UPS is crucial for maintaining protein homeostasis, DNA repair, and cell cycle regulation, which are essential for maintaining cellular function (3). Pathogens have evolved sophisticated strategies to hijack this system, particularly through manipulation of deubiquitinating enzymes (deubiquitinases; DUBs) (4).

*Salmonella*, an intracellular pathogen, engages unique mechanisms to survive in various cell types, including M cells, epithelial cells, macrophages, and dendritic cells (reviewed in reference 5). Although macrophages are central to the host’s innate immune defense, they paradoxically serve as a Trojan horse for *Salmonella* and support its replication (6–9). This phenomenon is facilitated by the bacterium’s ability to reprogram host cells such that it can survive within a specialized vacuole (10–12). The intracellular survival of *Salmonella* depends on effector proteins encoded from *Salmonella* pathogenicity islands 1 and 2 (SPI-1 and SPI-2), which manipulate host cell processes, immune responses, and cell signaling pathways, including those regulated by the UPS (reviewed in references 13, 14). Treating *Salmonella* infections is becoming progressively more challenging due to its rising Antimicrobial resistance (AMR) (1, 2), making it important to explore alternative therapeutic strategies. Host-targeted therapies (HTTs) offer a promising approach to control infection by disrupting the cellular pathways that pathogens rely on for survival, thereby reducing the selective pressure for antibiotic resistance (15, 16). This approach offers a potential solution to address the escalating AMR crisis (16). Given the central role of the UPS in regulating host-pathogen interactions, targeting specific components of this system, particularly DUBs, could effectively impair the ability of *Salmonella* to survive and replicate within host cells (17). This study evaluates the therapeutic potential of DUB inhibitors and other UPS modulators as a host-targeting approach to clearing bacteria that could bring innovative treatments against *Salmonella* and other bacterial pathogens (18).

DUBs, central to the UPS, are responsible for removing ubiquitin and ubiquitin-like modifiers from substrates, reversing post-translational modifications, modulating signaling pathways, and replenishing the pool of free ubiquitin necessary for ongoing ubiquitination processes (19). This diverse functionality impacts numerous cellular functions, including protein degradation, cellular localization, and protein-protein interactions. Through these mechanisms, DUBs influence a wide array of biological processes, including those related to human health, viral infections, and pathogen-host interactions (20–24). Notably, pathogenic bacteria, such as the intracellular pathogen *Salmonella*, exploit the host’s ubiquitination machinery to evade immune responses and establish persistent infections (18, 25). Targeting DUBs within the ubiquitin system presents a possible avenue for therapeutic development, potentially disrupting the ability of bacteria to survive and replicate within host cells (18).

In this study, we investigated the therapeutic potential of targeting the UPS as a host-directed strategy against bacterial infections. Using a curated library of UPS modulators and DUB inhibitors, we identified compounds that enhanced macrophage-mediated clearance of *Salmonella enterica* serovar Typhimurium. From this screen, the dual USP25/USP28 inhibitor AZ-1 emerged as a lead candidate. In a murine model of *Salmonella* infection, AZ-1 treatment reduced bacterial colonization, mitigated early weight loss, and improved clinical scores, but with limitations as a monotherapy in terms of improving survival. To validate target specificity, we performed USP25 knockdown in macrophages, which independently suppressed intracellular *Salmonella* replication, supporting USP25 as a functional host factor and possible drug target for host-targeted therapies against infections. Notably, AZ-1 also reduced the intracellular survival of ESKAPE pathogens, including *Klebsiella pneumoniae*, *Acinetobacter baumannii*, and *Pseudomonas aeruginosa*, demonstrating broader activity. These findings establish host DUBs, particularly USP25, as tractable targets for host-directed therapeutics and support further development of UPS-targeted strategies to combat intracellular bacterial infections.

## RESULTS

### Small-molecule modulators targeting the ubiquitin pathway as potential compounds enhancing macrophage-mediated bacterial clearance

To investigate the potential of targeting the UPS for host-directed antimicrobial therapy, we conducted a high-throughput screen (HTS) using a library of 257 small-molecule modulators that inhibit specific components of the UPS. Our goal was to identify compounds that enhance macrophage-mediated clearance of *Salmonella enterica* serovar Typhimurium without compromising host cell viability. In parallel, we assessed whether these compounds had direct antibacterial activity in axenic culture and measured their effects on host inflammatory signaling, including tumor necrosis factor alpha (TNF-α) production.

**Fig 1:**
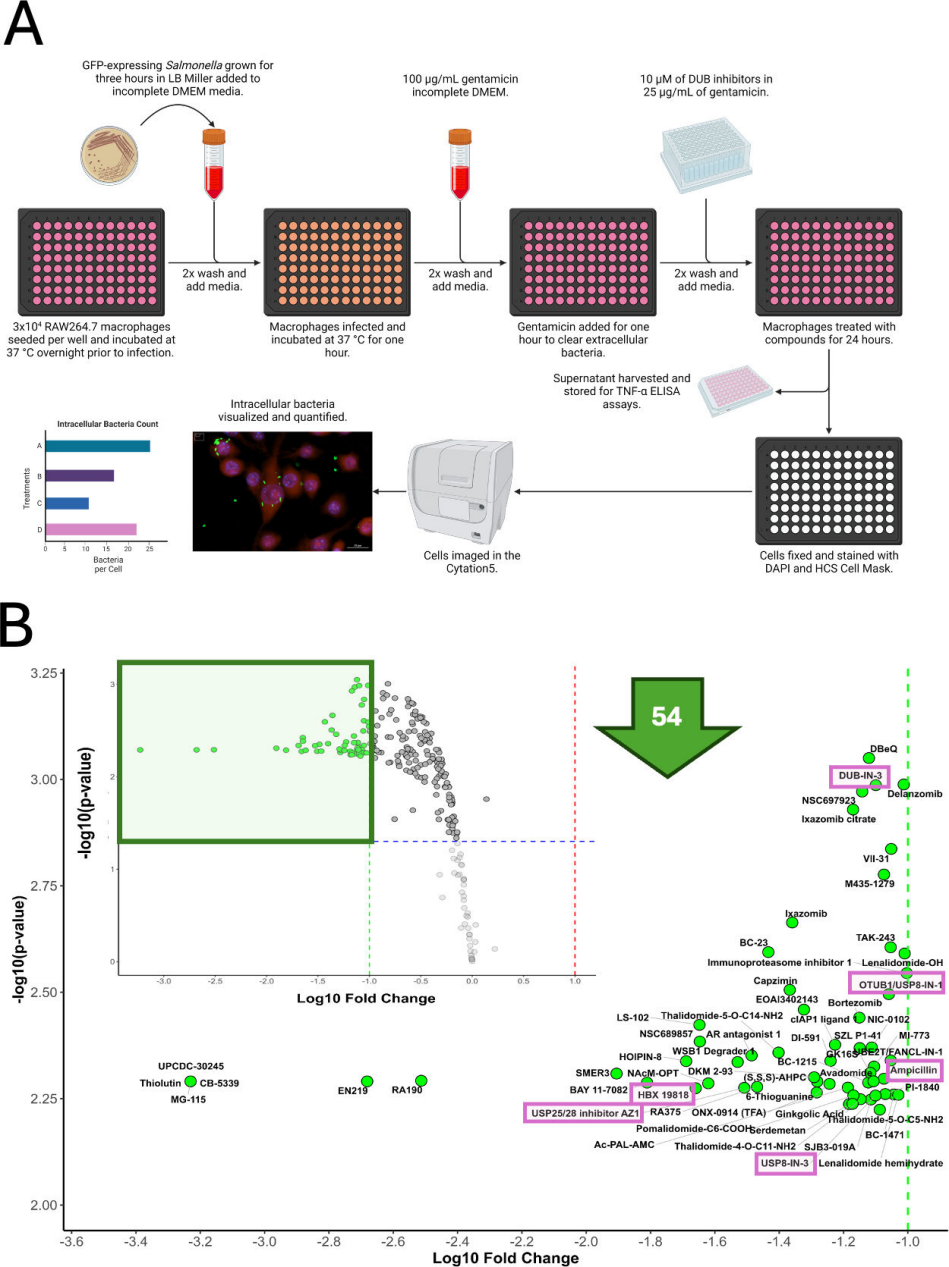
Screening of modulators of the UPS pathway for compounds supporting macrophage-mediated bacterial clearance. (**A**) Schematic representation of the high-throughput assay designed to identify compounds that enhance macrophage-mediated clearance of *Salmonella* Typhimurium strain UK-1 (multiplicity of infection [MOI] 30:1). The ubiquitination compound library (MedChem Express, HY-L050) was used for screening at a 10 µM concentration. This library comprises 257 small-molecule modulators targeting key enzymes in the ubiquitin pathway. (**B**) The impact of UPS pathway inhibitors on intracellular *Salmonella* was assessed. Intracellular bacterial counts were measured at 24 h post-infection with *Salmonella* (MOI 30:1) using an HTS assay and normalized to cell count. Data were analyzed in R, with mean replicate values calculated for each treatment. Fold changes and *P*-values compared to vehicle controls were determined using *t*-tests, followed by Benjamini-Hochberg correction for multiple comparisons. Significant modulators of *Salmonella* infection were visualized using a volcano plot, indicating the log10 fold change and −log10(*P*-value). DMEM, Dulbecco’s modified Eagle medium; LB, Lennox broth.

In the primary screen, macrophages infected with green fluorescent protein (GFP)-labeled *Salmonella* Typhimurium UK-1 were seeded into 96-well plates. Hoechst stain was used to label host cell nuclei, while HCS CellMask Red highlighted the cytoplasm, enabling precise quantification of intracellular bacteria while minimizing interference from extracellular bacteria (Fig. 1A). Following infection, cells were treated with each of the 257 small-molecule UPS-targeting compounds and incubated to allow intracellular bacterial uptake and modulation. High-content imaging was used to capture and quantify the number of intracellular bacteria per cell, evaluating the effect of each compound. The screen identified 59 compounds that significantly reduced intracellular bacterial burden compared to vehicle-treated controls (Fig. 1B; Table 1), with several compounds showing over 10-fold reductions and high statistical significance. Among these, the most potent hits—CB-5339, MG-115, Thiolutin, UPCDC-30245, EN219, RA190, SMER3, BAY 11-7082, HOIPIN-8, HBX 19818, LS-102, NSC689857, NAcM-OPT, WSB1 Degrader 1, and USP25/28 inhibitor AZ-1—achieved the largest reductions in bacterial load (≥1.5 log10 fold change).

**TABLE 1:** Effects of ubiquitin pathway-modulating compounds on macrophage-mediated bacterial clearance, cell number, and axenic growth^*a*^

Compound	Log10 FC (intracellular bacteria)	−Log10 *P*-value	Effect on nucleus count by minimum 50%	Effect on axenic growth by minimum 50%
(**S,S,S)-AHPC (hydrochloride**)	−1.28	2.29	–	–
**6-Thioguanine**	−1.24	2.28	–	–
**Ac-PAL-AMC**	−1.19	2.28	–	–
Ampicillin	−1.07	2.30	–	Decrease
**AR antagonist 1 (hydrochloride**)	−1.49	2.35	–	–
**Avadomide**	−1.11	2.29	–	–
**BAY 11-7082**	−1.81	2.29	–	–
**BC-1215**	−1.12	2.29	–	–
**BC-1471**	−1.04	2.26	–	–
BC-23	−1.43	2.59	Decrease	–
Bortezomib	−1.15	2.44	Decrease	Decrease
Capzimin	−1.37	2.51	Decrease	–
CB-5339	−3.23	2.29	Decrease	–
**cIAP1 ligand 1**	−1.23	2.38	–	–
**DBeQ**	−1.12	3.05	–	–
Delanzomib	−1.01	2.99	Decrease	–
**DI-591**	−1.24	2.34	–	–
**DKM 2-93**	−1.29	2.30	–	–
DUB-IN-3	−1.10	2.99	Decrease	–
**EN219**	−2.68	2.29	–	–
EOAI3402143	−1.32	2.46	Decrease	–
**Ginkgolic acid**	−1.15	2.25	–	–
**GK16S**	−1.12	2.31	–	–
HBX 19818	−1.66	2.27	Increase	–
HOIPIN-8	−1.69	2.34	Decrease	–
**Immunoproteasome inhibitor 1**	−1.00	2.54	–	–
Ixazomib	−1.36	2.66	Decrease	Decrease
Ixazomib citrate	−1.17	2.93	Decrease	Decrease
**Lenalidomide (hemihydrate**)	−1.03	2.26	–	–
**Lenalidomide-OH**	−1.01	2.59	–	–
LS-102	−1.65	2.42	Decrease	–
M435-1279	−1.07	2.78	Decrease	–
MG-115	−3.23	2.29	Decrease	–
**MI-773**	−1.10	2.33	–	–
**NAcM-OPT**	−1.62	2.29	–	–
NIC-0102	−1.11	2.37	Decrease	–
NSC689857	−1.65	2.38	Decrease	–
NSC697923	−1.14	2.97	Decrease	–
ONX-0914 (TFA)	−1.28	2.26	Decrease	–
OTUB1/USP8-IN-1	−1.06	2.50	–	–
**PI-1840**	−1.07	2.26	–	–
**Pomalidomide-C6-COOH**	−1.17	2.26	–	–
RA190	−2.51	2.29	Decrease	–
RA375	−1.47	2.28	Decrease	–
**Serdemetan**	−1.18	2.24	–	–
SJB3-019A	−1.11	2.25	Decrease	–
SMER3	−1.91	2.31	Decrease	–
**SZL P1-41**	−1.15	2.37	–	–
TAK-243	−1.05	2.61	Decrease	–
**Thalidomide-4-O-C11-NH2 (hydrochloride**)	−1.17	2.24	–	–
**Thalidomide-5-O-C14-NH2 (hydrochloride**)	−1.40	2.36	–	–
**Thalidomide-5-O-C5-NH2 (hydrochloride**)	−1.09	2.22	–	–
Thiolutin	−3.23	2.29	Decrease	Decrease
**UBE2T/FANCL-IN-1**	−1.05	2.34	–	–
UPCDC-30245	−3.23	2.29	Decrease	–
**USP25/28 inhibitor AZ-1**	−1.51	2.28	–	–
**USP8-IN-3**	−1.10	2.26	–	–
**VII-31**	−1.05	2.84	–	–
**WSB1 Degrader 1**	−1.53	2.34	–	–

^*a*^ Compounds that enhance bacterial clearance without inducing significant macrophage cytotoxicity are highlighted in bold for their therapeutic potential. "–,” not significant.

**Fig 2:**
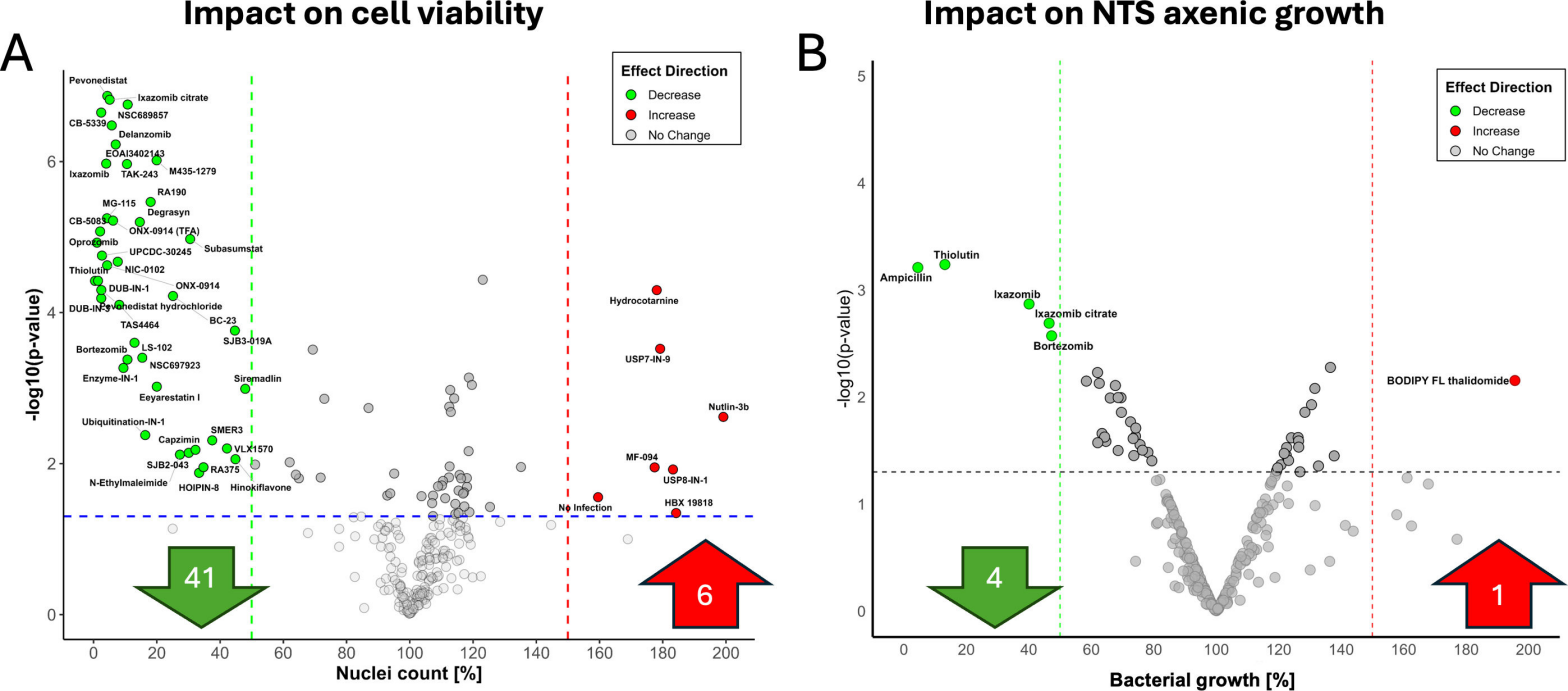
Screening of UPS pathway modulators for compounds that decrease cell count and inhibit bacterial growth in axenic culture. (**A**) An experiment was performed as in Fig. 1B. Nuclei count of macrophages treated with UPS pathway inhibitors at a concentration of 10 µM. The ubiquitination compound library (MedChem Express, HY-L050), comprising 257 small-molecule modulators targeting key enzymes in the ubiquitin pathway, was used for this screening. Data analysis was conducted in R, calculating the mean nuclei count for each treatment and comparing it to vehicle controls using *t*-tests (*n* = 4). (**B**) The effect of UPS pathway inhibitors on bacterial growth in axenic culture was assessed at 24 h post-infection, with compounds also used at a concentration of 10 µM. Data analysis in R involved calculating mean bacterial counts from triplicate samples (*n* = 3), determining fold changes, and assessing statistical significance using *t*-test analysis, with results visualized in a volcano plot to highlight significant modulators.

To ensure specificity for host-targeted activity, we next examined whether compound efficacy was associated with host cytotoxicity. Several compounds that reduced *Salmonella* burden also markedly decreased macrophage viability—evidenced by ≥50% reduction in nucleus count—and were therefore excluded as potential therapeutic candidates. These included bortezomib, capzimin, ixazomib citrate, delanzomib, EOAI3402143, MG-115, NIC-0102, NSC689857, and NSC697923. In contrast, compounds such as HBX 19818 reduced bacterial burden without affecting or even slightly improving host cell viability (Fig. 2A).

The axenic growth assay, designed to evaluate the direct antimicrobial activity of compounds in a *Salmonella*-only culture (free of host cells), revealed that several tested compounds could inhibit bacterial growth. Notably, bortezomib not only reduced the intracellular bacterial load but also directly inhibited *Salmonella* growth in axenic culture, reducing viability by at least 50%. Similarly, compounds such as thiolutin and ixazomib citrate demonstrated significant effects, reducing bacterial growth in both intracellular infection models and axenic cultures at a concentration of 10 µM (Fig. 2B). While these compounds hold promise as potential antimicrobials, their mechanisms of action might be independent of the UPS pathway, considering *Salmonella* lacks proteasomes. The antimicrobial properties of thiolutin are well-established (26). In contrast, AZ-1 and several other hits from the primary screen exhibited no growth-inhibitory effects in axenic culture, reinforcing their mechanism as host-targeted rather than directly bactericidal.

Compounds that reduced intracellular bacterial load without impairing host cell viability or affecting bacterial growth in axenic conditions (Table 2) included HBX 19818, OTUB1/USP8-IN-1, USP25/28 inhibitor AZ-1, USP8-IN-3, VII-31, and others. These molecules represent promising candidates for further development as host-directed therapeutics, capable of enhancing innate immune responses against intracellular pathogens without directly targeting the bacteria.

**TABLE 2:** Ubiquitin pathway-modulating compounds with a significant effect on macrophage-mediated bacterial clearance without affecting viability, and their targets within the UPS and other functions

Compound	Target/effect on ubiquitin pathway	Enzyme/details	Other known functions
HBX 19818	DUB inhibitor	USP7 (ubiquitin-specific peptidase 7)	
(S,S,S)-AHPC (hydrochloride)	Ubiquitin-proteasome system modulator	Modulates E3 ligase VHL	
6-Thioguanine	DUB inhibitor	Plpro, USP2	Antimetabolite affecting DNA/RNA synthesis
Ac-PAL-AMC	Fluorogenic substrate	Proteasome	
AR antagonist 1 (hydrochloride)	E3 ligase ligand binder	VHL	Androgen receptor antagonist
Avadomide	Cereblon modulator	Modulates cereblon, part of CRL4 E3 ubiquitin ligase	
BAY 11-7082	DUB inhibitor	USP7, USP21	IκB kinase inhibitor
BC-1215	E3 ligase inhibitor	F-box protein 3 (Fbxo3) inhibitor	
BC-1471	DUB inhibitor	STAM-binding protein (STAMBP) deubiquitinase	
cIAP1 ligand 1	IAP inhibitor	Inhibits cIAP1 (cellular inhibitor of apoptosis protein)	
DBeQ	Proteasome inhibitor	p97/VCP inhibitor	
DI-591	DUB inhibitor	USP14 (ubiquitin-specific peptidase 14)	
DKM 2-93	Ubiquitin-like modifier activating enzyme inhibitor	UBA5	
EN219	E3 ligase inhibitor	RNF114	
Ginkgolic acid	Proteasome inhibitor	Inhibits proteasome activity	
GK16S	DUB probe	UCHL1	
Immunoproteasome inhibitor 1	Immunoproteasome inhibitor	Inhibits immunoproteasome, affecting MHC class I pathway	
Lenalidomide (hemihydrate)	Cereblon modulator	Modulates cereblon, part of CRL4 E3 ubiquitin ligase	
Lenalidomide-OH	Cereblon modulator	Modulates cereblon, part of CRL4 E3 ubiquitin ligase	
MI-773	MDM2 inhibitor	Inhibits MDM2, affecting p53 ubiquitination	
NAcM-OPT	Cullin neddylation 1 (DCN1) inhibitor	DCN1-UBE2M interaction inhibitor	
OTUB1/USP8-IN-1	DUB inhibitor	Inhibits OTUB1 and USP8	
PI-1840	Selective chymotrypsin-like (CT-L) inhibitor	Unknown target	
Pomalidomide-C6-COOH	Cereblon modulator	Modulates cereblon, part of CRL4 E3 ubiquitin ligase	
Serdemetan	MDM2 inhibitor	Inhibits MDM2, affecting p53 ubiquitination	
SZL P1-41	E3 ligase inhibitor	Skp2 inhibitor	
Thalidomide-4-O-C11-NH2 (hydrochloride)	Cereblon modulator	Modulates cereblon, part of CRL4 E3 ubiquitin ligase	
Thalidomide-5-O-C14-NH2 (hydrochloride)	Cereblon modulator	Modulates cereblon, part of CRL4 E3 ubiquitin ligase	
Thalidomide-5-O-C5-NH2 (hydrochloride)	Cereblon modulator	Modulates cereblon, part of CRL4 E3 ubiquitin ligase	
UBE2T/FANCL-IN-1	E2 ubiquitin-conjugating enzyme inhibitor	Inhibits UBE2T and FANCL (Fanconi anemia pathway)	
USP25/28 inhibitor AZ-1	DUB inhibitor	Inhibits USP25 and USP28	
USP8-IN-3	DUB inhibitor	Inhibits USP8 (ubiquitin-specific peptidase 8)	
VII-31	NEDDylation pathway activator	Unknown target	
WSB1 degrader 1	E3 ligase inhibitor	Targets WSB1 (WD repeat and SOCS box containing protein 1)	

### Effect of the ubiquitin pathway inhibitors on TNF-α release from infected cells

Next, we evaluated the impact of selected DUB inhibitors and other UPS modulators on TNF-α secretion in RAW 264.7 macrophages infected with *Salmonella* (Fig. S1; Table S1). TNF-α, a key pro-inflammatory cytokine released by infected macrophages, was quantified using enzyme-linked immunosorbent assay (ELISA) at 24 h post-infection (hpi). The vehicle control (VC), infected with *Salmonella*, served as the baseline (100%) for comparing changes in TNF-α levels. A range of responses was observed, with some inhibitors significantly increasing TNF-α secretion, while others reduced it below baseline levels (Fig. S1). In total, 84 compounds significantly altered TNF-α secretion by at least 20%, either upregulating or downregulating its levels (Table S1), but 67 compounds were significant without altering cell viability (Fig. S1). Among the compounds that increased TNF-α, the USP7/USP47 inhibitor had the most pronounced effect, nearly tripling TNF-α levels. Similarly, USP18-IN-3, ML364, and HB007 almost doubled TNF-α levels, while USP9X-IN-1 resulted in a significant 30% increase. These compounds highlight potential pathways for enhancing inflammatory responses, which could be beneficial in specific therapeutic contexts but may also pose risks of physiological damage. Conversely, several compounds significantly decreased TNF-α levels. Notable examples include (S,R,S)-AHPC-Boc, which reduced TNF-α by 35%, ALV1 by 42%, and ALV2 by a substantial 53%. Additionally, AR antagonist 1 (hydrochloride) and alloxan (hydrate) decreased TNF-α by 34% and 25%, respectively. These findings identify potential candidates for mitigating hyperinflammatory conditions. Interestingly, some compounds that modified TNF-α levels, such as AR antagonist 1 (hydrochloride), BC-1215, DBeQ, MI-773, Thalidomide-5-O-C14-NH2 (hydrochloride), and cIAP1 ligand 1, also demonstrated the capacity to reduce infection in RAW 264.7 macrophages.

### Alterations in DUB transcript levels post-*Salmonella* infection

**Fig 3:**
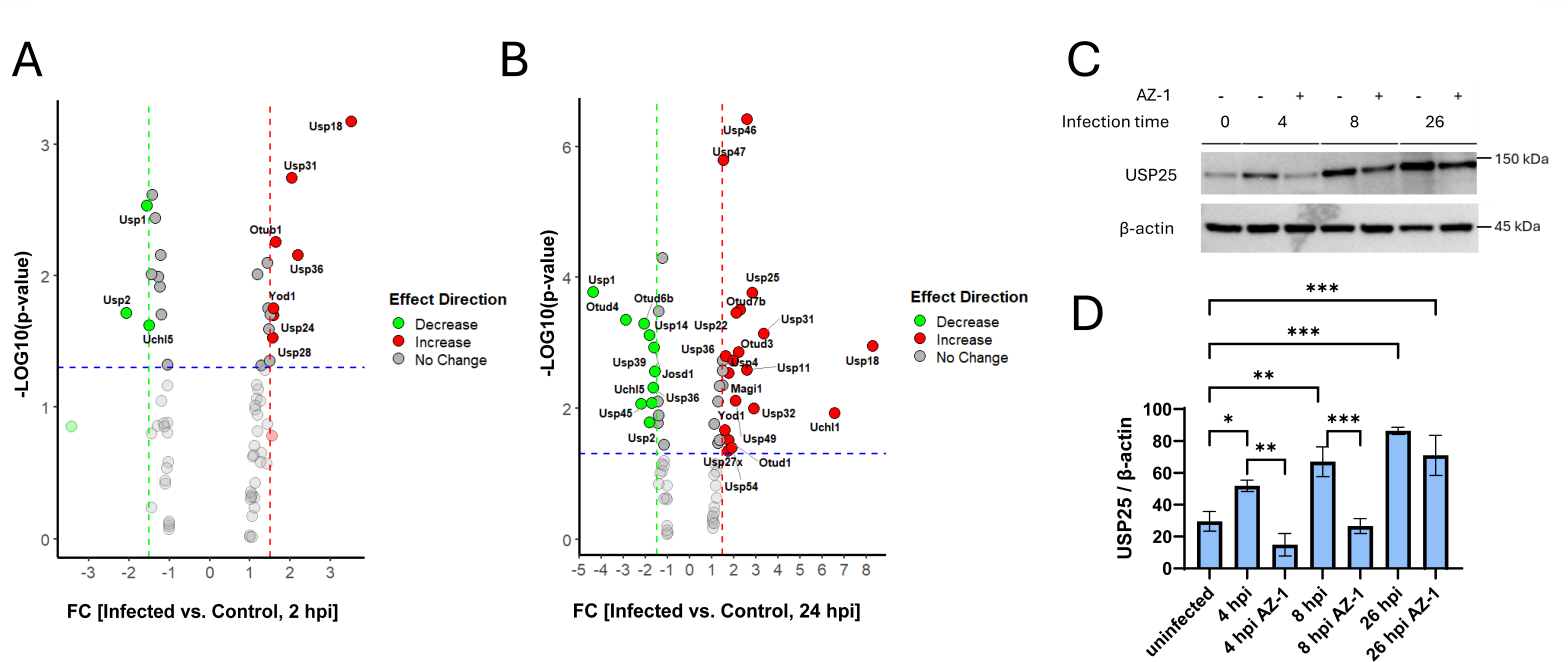
Regulation of DUB expression during *Salmonella* infection in murine macrophages. (**A and B**) Quantitative PCR profiling of DUB transcripts in RAW 264.7 macrophages infected with *Salmonella enterica* serovar Typhimurium strain UK-1 (multiplicity of infection 30:1). Cells were harvested at 2 h (**A**) and 24 h (**B**) post-infection. Total RNA was extracted, reverse transcribed, and analyzed using a PrimePCR DUB-specific array. Data represent relative expression (log2 fold change) compared to uninfected controls, analyzed in R from three biological replicates. (**C**) Western blot analysis of USP25 protein levels in RAW 264.7 macrophages infected with *Salmonella* for 0, 4, 8, or 26 h, in the presence or absence of AZ-1 (10 µM). β-Actin served as a loading control. AZ-1 treatment suppressed infection-induced upregulation of USP25, suggesting its effect on host DUB signaling during infection. (**D**) Densitometric quantification of USP25 expression during infection with *Salmonella* in the presence or absence of AZ-1 inhibitor, normalized to β-actin using ImageJ. Statistical analysis: one-way analysis of variance was used to compare time points and treatment groups. *n* = 3 independent experiments. Data are presented as mean ± SEM. *P < 0.05, **P < 0.01, ***P < 0.001.

Since many of the UPS modulators were inhibitors of DUBs, we next analyzed the transcript levels of DUBs in RAW 264.7 macrophages following infection with the laboratory strain of *Salmonella* Typhimurium. Quantitative real-time PCR was employed to assess the expression changes in 70 DUBs between *Salmonella*-infected and non-infected RAW 264.7 macrophages at 2 and 24 hpi (Fig. 3). At the 2 hpi time points, three DUB transcripts were significantly upregulated, while seven were significantly downregulated (Fig. 3A). By 24 hpi, the number of significantly upregulated DUBs increased to 10, while 19 DUBs showed significant downregulation (Fig. 3B). At 2 hpi, *Usp2, Usp1*, and *Uchl5* were downregulated, while *Usp28*, *Usp24, Yod1, Otub1, Usp31, Usp36,* and *Usp18* were upregulated. Notably, DUBs such as *Otud1, Otud3, Otud7b, Uchl1, Usp11, Usp18, Usp22, Usp25, Usp27x, Usp31, Usp32, Usp36, Usp4, Usp46, Usp47, Usp49, Usp54*, and *Yod1* exhibited substantial increases in expression at 24 hpi, suggesting their potential involvement in the macrophage response to *Salmonella* infection. Conversely, DUBs including *Josd1, Otud4, Otud6b, Uchl5, Usp1, Usp14, Usp2, Usp36, Usp39*, and *Usp45* were among those downregulated at 24 hpi, indicating a potential suppression of their expression during infection. Interestingly, *Usp18, Usp31, Usp36*, and *Yod1* were consistently upregulated at both time points (2 and 24 hpi), suggesting a sustained role in the macrophage response to *Salmonella* infection. In contrast, *Usp1, Usp2*, and *Uchl5* remained downregulated across both time points, indicating a prolonged suppression during infection. To validate transcript-level findings, we examined the protein expression of one key DUB, USP25, at later infection times because protein synthesis often lags behind transcriptional changes. Consistent with transcript data, USP25 protein levels increased progressively, with strong induction by 26 hpi and detectable elevation as early as 4 hpi (Fig. 3C and D), supporting its infection-responsive regulation. While a reduction in USP25 protein was observed in AZ-1–treated samples at 4hpi and 8 hpi, this may reflect indirect effects, such as decreased bacterial burden or altered host signaling. These findings highlight the dynamic regulation of DUBs during *Salmonella* infection and suggest a critical role for specific DUBs, such as USP25, in the macrophage response.

Using published data sets, we analyzed the modulation of DUBs in monocyte-derived dendritic cells (moDCs) infected with three distinct *Salmonella* strains: Ty2 (*Salmonella* serovar Typhi), D23580 (*Salmonella* serovar Typhimurium, clinical isolate, invasive non-typhoidal *Salmonella* strain, iNTS), and LT2 (*Salmonella* serovar Typhimurium, laboratory strain, NTS) at 6 hpi (27). RNA sequencing of *Salmonella*-infected moDCs, sorted via fluorescence-activated cell sorting at 6 hpi, revealed significant differential gene expression between infected and uninfected cells in terms of DUB expression levels. At 6 hpi with the Ty2 strain, several DUB transcripts were significantly regulated. *Otud1, Otud7b, Usp11, Usp18*, and *Usp25* exhibited significant upregulation, while *Otud6b* and *Otud3* were downregulated, though *Otud3* was upregulated in our study (Fig. S2A). Similarly, infection with the D23580 strain resulted in the upregulation of *Otud1, Otud7b, Usp11, Usp18,* and *Usp25* at 6 hpi, while *Otud6b* was downregulated (Fig. S2B). In moDCs infected with the LT2 strain, *Otud1, Otud7b, Usp11, Usp18, Usp25*, and *Usp36* exhibited substantial increases in expression at 6 hpi (28). Conversely, DUBs such as *Otud6b, Usp39,* and *Usp45* were downregulated, along with Otud3, which was significantly upregulated in our study (Fig. S2C). A time-course RNA-seq analysis of DUB expression in LT2-infected moDCs at 2, 4, and 6 hpi (Fig. S2D) revealed that *Usp25, Usp18, Usp11*, and *Usp41* increased at later stages, suggesting roles in sustaining the immune response, while *Otud4* and *Uchl5* decreased, indicating early regulatory functions.

Collectively, the differential expression analysis of DUBs in response to diverse Salmonella strains—including typhoidal, non-typhoidal, and invasive strains (Ty2, D23580, and LT2)—as well as in RAW 264.7 macrophages infected with a laboratory strain of *S.* Typhimurium revealed consistent regulatory patterns. Across all these studies, DUBs such as *Otud1, Otud7b, Usp11, Usp18*, and *Usp25* were significantly upregulated, while *Otud6b* was consistently downregulated.

### Efficacy of AZ-1 in promoting clearance of intracellular *Salmonella*

**Fig 4:**
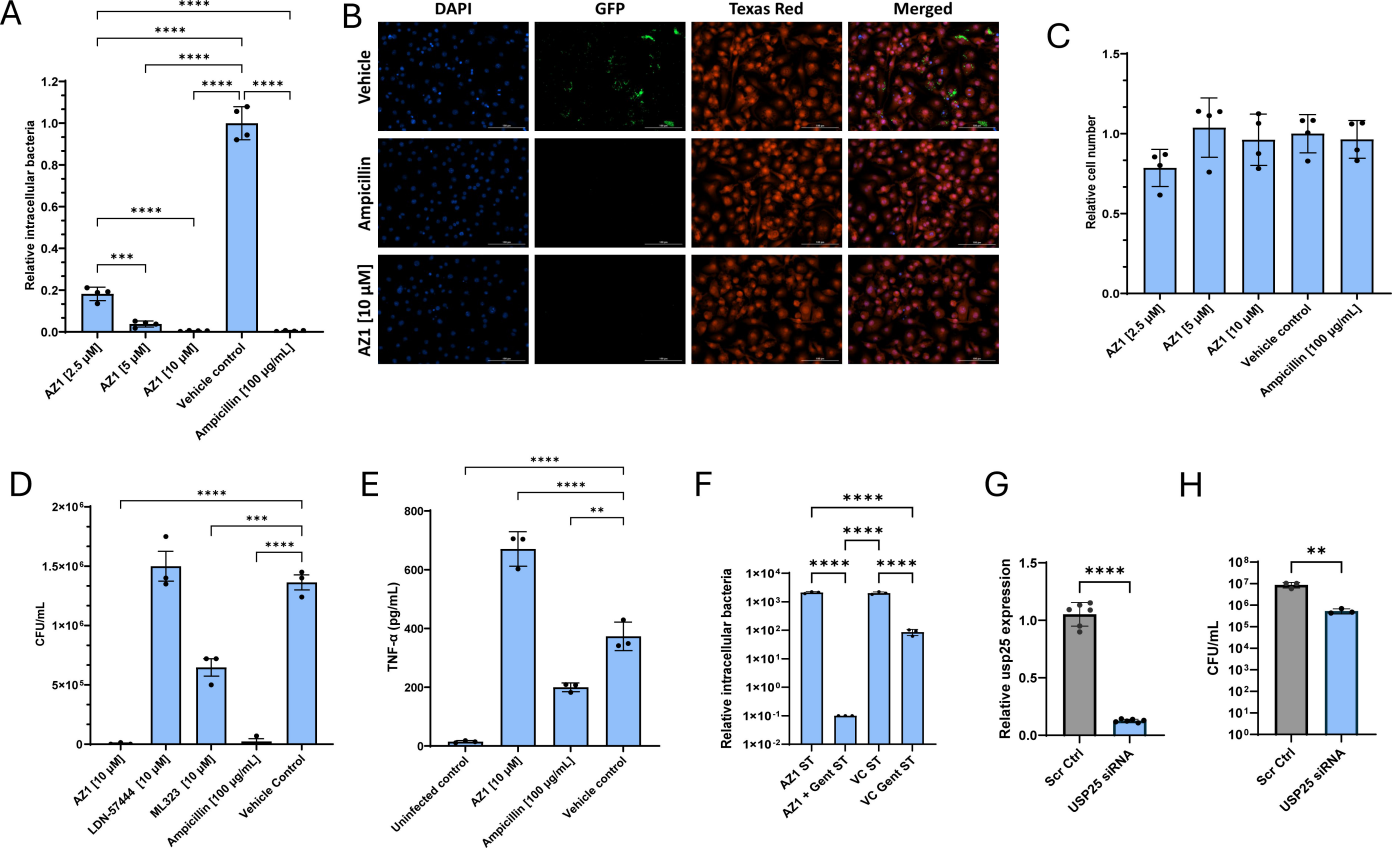
AZ-1 (USP25/28 inhibitor) effectively clears *Salmonella* in host cells. (**A–C**) Representative images and quantification of *Salmonella enterica* serovar Typhimurium strain UK-1-infected RAW 264.7 macrophages treated with AZ-1 (10 µM), ampicillin (100 µg/mL), or vehicle control (dimethyl sulfoxide [DMSO]) for 26 h post-infection (multiplicity of infection 30:1). (**A**) Hoechst staining (blue) labels nuclei, while GFP fluorescence (green) indicates intracellular *Salmonella*. (**B**) Merged images show Hoechst (blue), GFP-*Salmonella* (green), and CellMask (red) to highlight cell structure. (**C**) Quantification of infected cells, comparing treatments. (**D**) Intracellular bacterial survival was assessed by gentamicin protection assay followed by colony-forming unit (CFU) enumeration, comparing AZ-1 to other UPS inhibitors (LDN-57444 and ML-323), ampicillin, and vehicle control. (**E**) TNF-α secretion was measured in supernatants 24 h post-infection to assess inflammatory responses following treatment with AZ-1 or ampicillin. (**F**) To examine the impact of AZ-1 in the presence or absence of extracellular bacterial killing, infected macrophages were treated with gentamicin (100 µg/mL) for 1 h post-infection, followed by AZ-1 treatment alone, AZ-1 plus low-dose gentamicin ( 25 µg/mL), or respective vehicle controls. Intracellular bacteria were quantified by GFP fluorescence. (**G**) Quantitative reverse transcription PCR analysis of USP25 following 48 h small interfering RNA (siRNA) transfection. A scrambled negative control siRNA was used for comparison of USP25 relative expression. (**H**) Intracellular bacterial survival was quantified using a gentamicin protection assay followed by CFU counts, evaluating the effect of USP25 siRNA, with the scrambled control serving as a negative reference. Statistical analysis: one-way analysis of variance with Tukey’s multiple comparisons test was used for panels C, D, F. Unpaired two-tailed *t*-tests were used for panels E, G, H. Data are mean ± SEM from three independent experiments (*n* = 3). *P < 0.05, **P < 0.01, ***P < 0.001, ****P < 0.0001.

The consistent upregulation of *Usp25* in macrophages and moDCs during *Salmonella* infection, coupled with its identification in a high-throughput screen as a standout target, led us to evaluate AZ-1, a USP25/28 inhibitor. AZ-1 significantly reduced intracellular *Salmonella* without affecting host cell viability (Fig. 1). Its ability to enhance macrophage-mediated bacterial clearance consistently and safely highlights its strong potential for further development. In addition to AZ-1, we tested USP1 and UCH-L1 inhibitors, as these DUBs were differentially expressed during infection (Fig. 4).

AZ-1 significantly reduced intracellular bacterial counts in a dose-dependent manner, with maximal activity observed at 10 μM, comparable to the efficacy of ampicillin (Fig. 4A and B). Importantly, cell viability remained unaffected at all concentrations tested (Fig. 4C), and no cytotoxicity was detected in uninfected cells treated with AZ-1 (Fig. S3). Colony plating further confirmed that AZ-1 significantly reduced intracellular bacterial counts compared to vehicle controls and other tested DUB inhibitors (Fig. 4D). In addition to its antimicrobial effects, AZ-1 also significantly upregulated TNF-α secretion during *Salmonella* infection (Fig. 4E), suggesting it may modulate the immune response in a beneficial way.

To investigate the role of gentamicin in facilitating AZ-1’s intracellular activity, we conducted an experiment where RAW 264.7 macrophages were infected with *Salmonella* Typhimurium for 1 h. After the infection, extracellular bacteria were eliminated by applying gentamicin (100 μg/mL) for an additional hour, followed by its removal. Cells were then treated for the remainder of the infection period with either AZ-1 alone, AZ-1 combined with a lower concentration of gentamicin (25 μg/mL), vehicle control, or vehicle control with gentamicin. The results showed that AZ-1 alone did not reduce the intramacrophage bacterial load in the absence of gentamicin, while the combination of AZ-1 with gentamicin led to a significant 4-log reduction in bacteria counts (Fig. 4F). This indicates that AZ-1’s efficacy requires effective suppression of extracellular bacteria by another means.

To exclude the possibility that gentamicin alone contributes to intracellular bacterial clearance over prolonged periods, we evaluated various sequential treatment regimens after 1 h of infection with *Salmonella* of RAW 264.7 macrophages. Specifically, we used an initial high concentration of gentamicin (applied for 1 h) to eliminate extracellular bacteria, followed by lower sustained concentrations during the overnight incubation. The tested regimen included the following initial/sustained gentamicin concentration pairs (in micrograms per milliliter): 100/25, 50/5, 25/2.5, and 100/20. These regimens were combined with either vehicle control or AZ-1 treatment. In all conditions, AZ-1 consistently reduced bacterial load after overnight infection, reinforcing its efficacy when extracellular bacteria are effectively controlled by gentamicin (Fig. S4). By testing multiple gentamicin dosing strategies, we confirmed that prolonged gentamicin exposure alone does not significantly influence the clearance of intracellular bacteria. Instead, the observed reduction in bacterial loads is attributed to AZ-1’s intracellular activity, which depends on the suppression of extracellular bacteria during the initial infection period. Importantly, AZ-1 did not affect *Salmonella* growth under axenic conditions (Fig. S5).

To further evaluate the role of USP25 in intracellular bacterial survival, we generated USP25 knockdown RAW 264.7 macrophages using small interfering RNA (siRNA) (Fig. 4G). Compared to cells treated with scrambled siRNA, USP25-deficient macrophages exhibited a significant reduction in intracellular *Salmonella* burden at 24 hpi (*P* < 0.01), confirming that USP25 promotes bacterial persistence (Fig. 4H). These results validate our screening findings and highlight USP25 as a potential target for host-directed or adjunct antimicrobial therapies. Together, these findings validate USP25 as a viable target for host-directed therapy and support AZ-1 as a promising candidate for combination approaches with antibiotics like gentamicin. By promoting macrophage-mediated bacterial clearance, preserving host cell viability, and enhancing inflammatory responses, AZ-1 offers a host-targeted strategy to combat persistent intracellular *Salmonella* infection. Although the precise mechanisms through which AZ-1 limits intramacrophage bacterial growth remain to be elucidated, its robust intracellular activity warrants further investigation.

**Fig 5:**
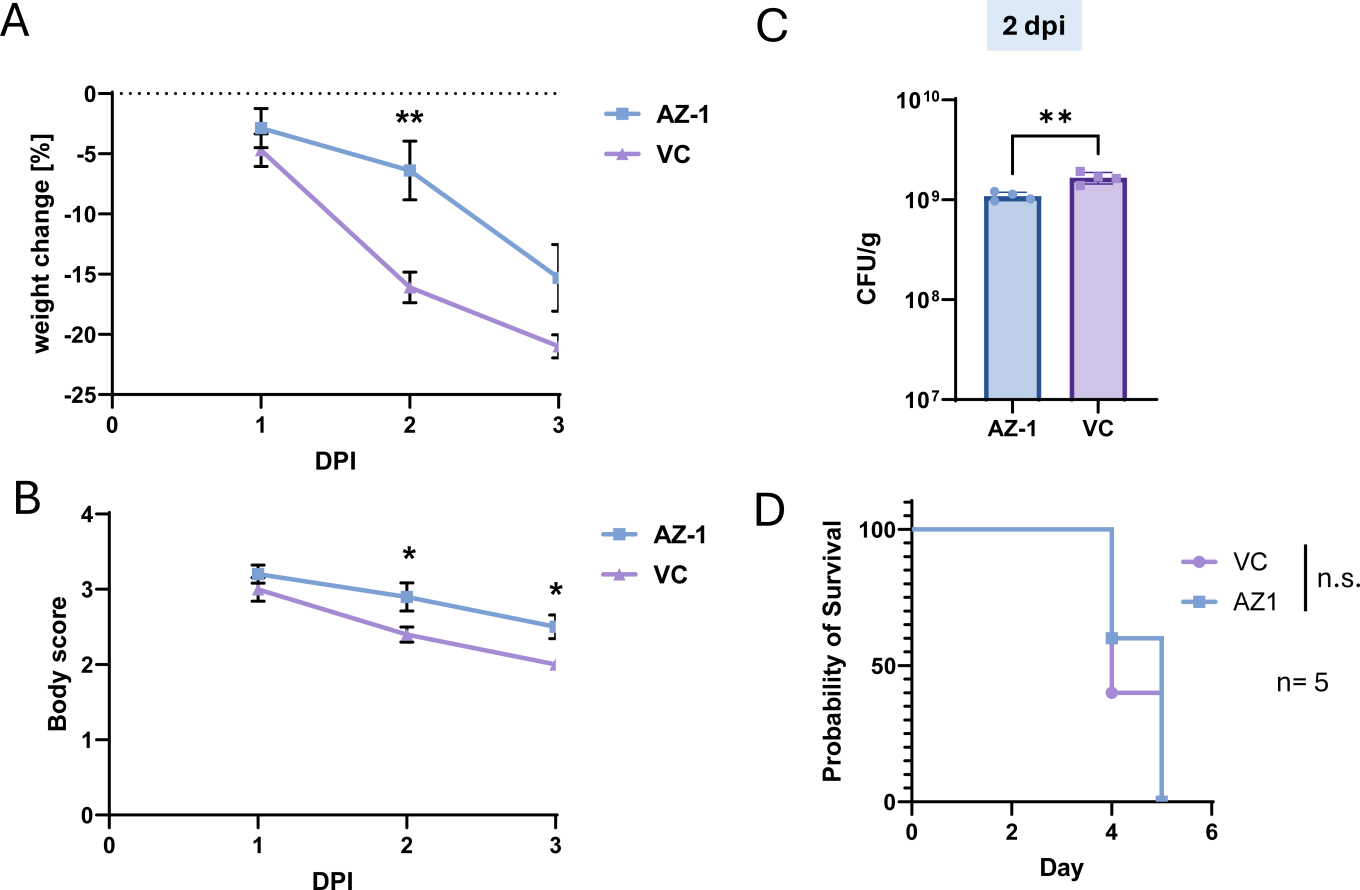
Treatment of C57BL/6J mice with AZ-1 attenuates weight loss and disease severity but does not significantly prolong survival. (**A**) C57BL/6J mice were infected with *Salmonella enterica* serovar Typhimurium and treated with AZ-1 (20 mg/kg, oral gavage) or VC starting on day 1 post-infection. Body weight was measured daily to assess infection severity. (**B**) Clinical disease scores were recorded daily to monitor progression of infection symptoms. (**C**) Fecal bacterial loads were quantified at 2 days post-infection (dpi) by plating stool samples on selective agar and counting colony-forming units (CFUs). (**D**) Survival of infected mice was monitored over the course of infection following treatment with AZ-1 or vehicle control. Statistical analysis: two-way analysis of variance (ANOVA) with Šídák’s correction was used for panel **A**, and two-way repeated measures ANOVA with Bonferroni’s test for panel **B**. An unpaired *t*-test was used for panel **C**, and log-rank (Mantel-Cox) test for panel **D**. Data are shown as mean ± SEM.*P < 0.05, **P < 0.01.

### *In vivo* evaluation of AZ-1 reveals partial efficacy in reducing *Salmonella* infection severity in a murine model

To assess the *in vivo* efficacy of AZ-1, we used a streptomycin-pretreated murine model of *Salmonella* infection. Streptomycin was administered to disrupt the gut microbiota and enhance *Salmonella* colonization (29). Mice were treated with AZ-1 (20 mg/kg) or VC starting at 1 day post-infection (dpi). AZ-1 treatment attenuated weight loss: while there was no significant difference in weight loss between AZ-1- and VC-treated mice at 1 dpi, mice receiving AZ-1 exhibited significantly reduced weight loss by 2 dpi (*P* = 0.0070) (Fig. 5A). This suggests a measurable protective effect of AZ-1 during infection progression. Body condition scores were also significantly improved in AZ-1-treated mice. Two-way repeated measures analysis of variance (ANOVA) revealed a significant effect of treatment (*P* = 0.0337) and time (*P* < 0.0001), and *post hoc* testing confirmed significantly higher scores at both 2 and 3 dpi (*P* < 0.05), indicating reduced clinical disease severity (Fig. 5B). Importantly, AZ-1 treatment significantly reduced fecal bacterial load at 2 dpi, as measured by colony-forming unit (CFU) enumeration (*P* < 0.005), suggesting enhanced early bacterial clearance or reduced colonization (Fig. 5C). Survival outcomes were also unaffected by AZ-1 monotherapy. Kaplan-Meier survival analysis showed no significant difference between AZ-1 and VC groups (log-rank *P* = 0.5485), and median survival differed only modestly (5 dpi vs 4 dpi, respectively) (Fig. 5D).

Together, these results indicate that AZ-1 partially mitigates disease severity and reduces early bacterial burden but fails to extend survival when administered as monotherapy. These findings are consistent with *in vitro* observations suggesting that AZ-1 may require combination therapy to achieve full therapeutic efficacy against *Salmonella*.

### Effect of AZ-1 inhibitor on the ERK1/2 and NF-κB signaling

**Fig 6:**
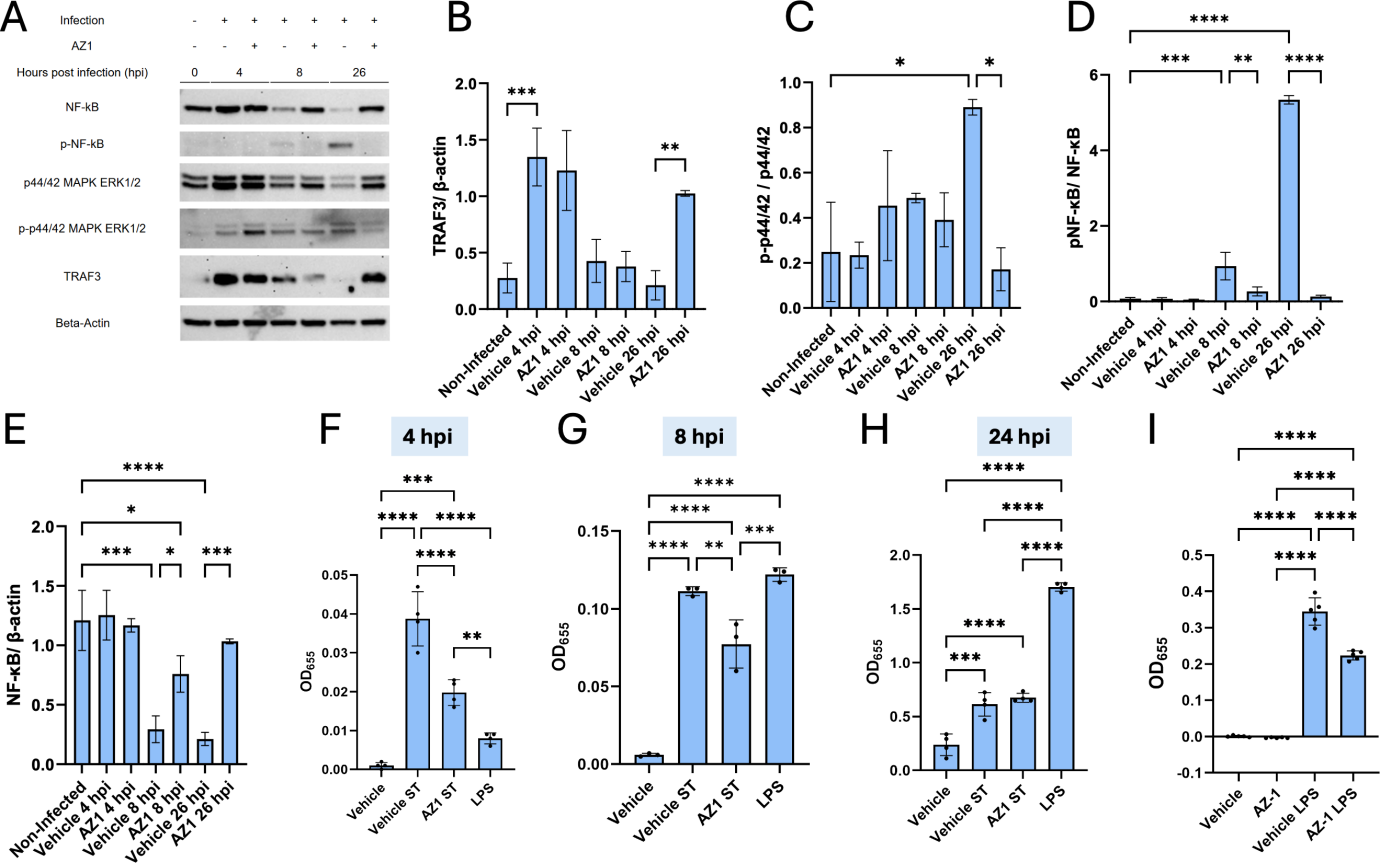
AZ-1 affects NF-κB signaling. (**A–E**) RAW 264.7 macrophages were infected with *Salmonella enterica* serovar Typhimurium strain UK-1 (multiplicity of infection 30:1) and treated with gentamicin (100 µg/mL) for 1 h to eliminate extracellular bacteria. Cells were then washed and cultured in the presence of AZ-1 (10 µM) or vehicle control (dimethyl sulfoxide, DMSO). Cell lysates were collected at 0 (uninfected), 4, 8, and 26 h post-infection and analyzed by Western blot (**A**) for NF-κB, phosphorylated NF-κB (p-NF-κB), ERK1/2 (p44/42 MAPK), phosphorylated ERK1/2 (p-p44/42), TRAF3, USP25, and β-actin. Densitometric quantification was performed using ImageJ to calculate signal intensity ratios for TRAF3/β-actin (**B**), p-p44/42/p44/42 (**C**), p-NF-κB/NF-κB (**D**), and NF-κB/β-actin (**E**). β-Actin blot was reused from Fig. 3D as they were part of the same internally controlled experiment. (F–H) A parallel infection model was conducted using RAW 264.7-Blue NF-κB reporter cells. Supernatants were collected at 4 (**F**), 8 (**G**), and 26 hpi (**H**) to measure NF-κB-driven secreted alkaline phosphatase activity. (**I**) RAW 264.7-Blue cells were treated with 500 ng/mL lipopolysaccharide (LPS) for 6 h in the presence of AZ-1 or DMSO vehicle control to assess AZ-1′s effect on Toll-like receptor 4 (TLR4)-mediated NF-κB activation (*n* = 5). Statistical analysis: one-way ANOVA followed by Tukey’s multiple comparisons test was used for panels A–E and I. Panels F–H were analyzed using unpaired two-tailed Student’s *t*-tests. Data are presented as mean ± SEM from three independent experiments (*n* = 3) unless otherwise noted. *P < 0.05, **P < 0.01, ***P < 0.001, ****P < 0.0001.

*Salmonella* infection is associated with alterations in host signaling pathways, including extracellular signal-regulated kinase 1/2 (ERK1/2) (30) and NF-κB, which are key regulators of immune responses and inflammation. Several *Salmonella* virulence factors are known to interfere with the NF-κB pathway (31–33). Understanding how these pathways are modulated during infection, as well as how potential therapeutic agents like AZ-1 influence these dynamics, could uncover novel mechanisms to mitigate bacterial pathogenesis. We analyzed the temporal dynamics of TNF receptor-associated factor 3 (TRAF3) expression, ERK1/2 activation, and NF-κB signaling during *Salmonella* infection with or without AZ-1 treatment. TRAF3, a known negative regulator of the non-canonical NF-κB pathway, was upregulated during the early stages of infection (4 hpi) in response to *Salmonella* infection (Fig. 6A and B). In contrast, AZ-1 treatment significantly enhanced TRAF3 levels during the late stages of infection (26 hpi, 24 h post-AZ-1 treatment). Additionally, AZ-1 treatment reduced the p-ERK1/2 to ERK1/2 ratio at 26 hpi compared to vehicle-treated infected cells, indicating that AZ-1 attenuates ERK1/2 activation during the late stages of *Salmonella* infection. However, no significant effects were observed at earlier points (Fig. 6A and C).

*Salmonella* infection activated NF-κB signaling, as demonstrated by increased phosphorylation of NF-κB (p-NF-κB) in Western blots at both 8 hpi and 26 hpi (Fig. 6A and D). Importantly, AZ-1 treatment markedly downregulated NF-κB phosphorylation at these time points, suggesting diminished NF-κB activation. Interestingly, the total NF-κB expression was increased in AZ-1-treated cells at 8 hpi and 24 hpi (Fig. 6A and E). The effect of AZ-1 on NF-κB activation was quantitatively validated using the QUANTI-Blue assay, which measures NF-κB-driven secretion of alkaline phosphatase (SEAP) through colorimetric OD_655_ readings. AZ-1-treated cells exhibited significantly reduced NF-κB activity compared to vehicle-treated controls at 4 hpi and 8 hpi, aligning with the Western blot findings. However, these differences were not observed at 24 hpi (Fig. 6F through H). A similar trend was observed in lipopolysaccharide (LPS)-treated cells, where AZ-1 also decreased LPS-induced NF-κB activation following 6 h of LPS treatment (Fig. 6I).

In conclusion, AZ-1 treatment modulates NF-κB and ERK1/2 signaling during *Salmonella* infection, emphasizing a role for AZ-1 in targeting host immune signaling pathways, potentially contributing to enhanced bacterial clearance.

### Efficacy of AZ-1 in promoting clearance of other bacterial infections

**Fig 7:**
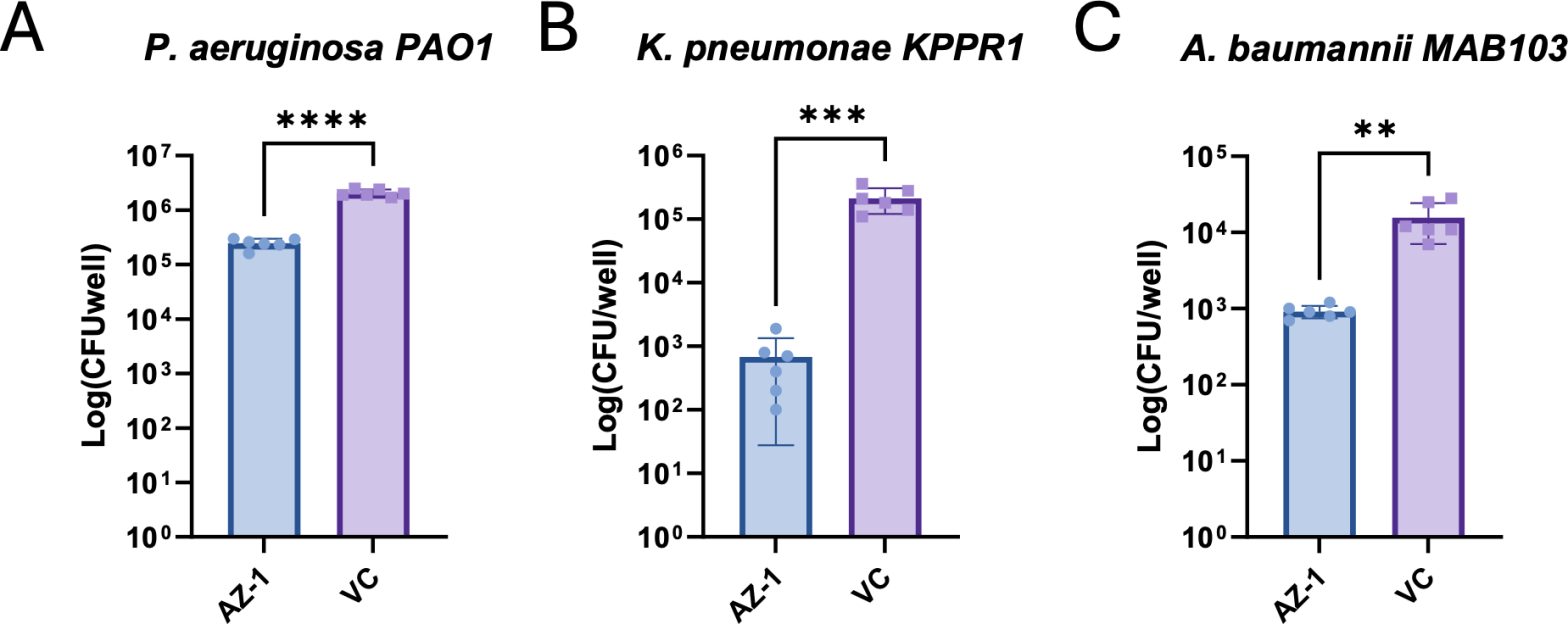
Effects of AZ-1 on the intramacrophage growth of various ESKAPE pathogens. RAW 264.7 macrophages were infected with (A) *Pseudomonas aeruginosa*, (B) *Klebsiella pneumoniae*, or (C) *Acinetobacter baumannii* at an MOI of 10. Following infection, extracellular bacteria were removed by gentamicin treatment, and cells were subsequently treated with AZ-1 (10 µM) or vehicle control (DMSO). After incubation, macrophages were lysed, and intracellular bacterial loads were quantified by CFU enumeration. Statistical analysis was performed using unpaired two-tailed Student’s *t*-tests. Bars represent the mean CFU ± SEM from six biological replicates (*n* = 6). The experiment was repeated three times, and a representative data set is shown. *P < 0.05, **P < 0.01, ***P < 0.001, ****P < 0.0001.

Subsequently, we tested the efficacy of AZ-1 on the clearance of other pathogens, including ESKAPE pathogens. ESKAPE pathogens—*Enterococcus faecium, Staphylococcus aureus, Klebsiella pneumoniae, Acinetobacter baumannii, Pseudomonas aeruginosa*, and *Enterobacter* species—are a group of antibiotic-resistant bacteria known for causing severe hospital-acquired infections (34). RAW 264.7 cells were infected with *P. aeruginosa* PAO1*, K. pneumoniae* KPPR1*,* and *A. baumannii* MAB103 at a multiplicity of infection (MOI) of 10. The infected macrophages were treated with AZ-1 or dimethyl sulfoxide (DMSO; control). After 4 h of treatment, the cells were lysed, and the bacterial load was determined by plating the lysates for CFU enumeration. The results demonstrate a significant reduction in CFUs for all three tested ESKAPE pathogens in the AZ-1-treated samples compared to the control, indicating the efficacy of AZ-1 in promoting clearance of the ESKAPE pathogens (Fig. 7). Testing AZ-1 and HBX 19818 on the intramacrophage growth of another gram-negative bacterium, *Francisella novicida*, which has a different intracellular lifestyle, evaluated using immortalized murine bone marrow-derived macrophages and RAW 264.7 cells indicated that AZ-1 and HBX 19818 had no effect on the growth of *F. novicida* within macrophages in the tested conditions (Fig. S6).

These findings show the importance of testing such novel host-targeting inhibitors across various bacterial pathogens and demonstrate the potential of the tested drugs as therapeutic agents for infections caused by *Salmonella*, *Pseudomonas*, *Klebsiella*, and *Acinetobacter*, but not *Francisella*.

## DISCUSSION

The escalating crisis of antibiotic resistance among bacterial pathogens necessitates innovative therapeutic approaches. This study demonstrates the potential of HTTs to combat infections by focusing on DUBs and their role in macrophage responses to *Salmonella*. Targeting host pathways rather than pathogens directly offers distinct advantages, particularly against bacteria like *Salmonella* and ESKAPE pathogens, which frequently resist conventional antibiotics (34).

High-throughput screening of a UPS modulator compound library revealed several promising candidates that significantly enhance bacterial clearance and modulate inflammatory responses without compromising host cell viability. Among them, DUB inhibitors were particularly effective, targeting key enzymes like USP7, USP8, and USP25 to disrupt *Salmonella*’s immune evasion mechanisms. Proteasome inhibitors such as DBeQ and ginkgolic acid also exhibited significant activity, which is known to be acting through ubiquitin-dependent and autophagic pathways (35–40). Additionally, CRBN modulators, including avadomide and lenalidomide derivatives, here reduced bacterial loads likely by targeting the CRL4 E3 ubiquitin ligase complex (41). MDM2 inhibitors (e.g., MI-773, serdemetan) effectively combat intracellular *Salmonella* by modulating the MDM2-p53 axis, critical for host defense (42–47). Beyond *Salmonella*, MDM2 is important in controlling outcomes of other infections, such as *Chlamydia* (48) and *Helicobacter pylori*, where it regulates p53-mediated host responses (49). The potential to disrupt *Salmonella* effector AvrA-mediated p53 stabilization presents a novel therapeutic avenue (50). Evaluating MDM2 inhibitors’ impact on AvrA-mediated virulence and p53 stabilization could provide new therapeutic strategies. Similarly, neddylation inhibitors (e.g., NAcM-OPT, VII-31) modulate immune responses by influencing cytokine production and inflammatory signaling in macrophages and neutrophils. Their ability to reduce pro-inflammatory cytokines, such as TNF-α and interleukin-6 (IL-6), and regulate the NF-κB pathway highlights their dual potential in limiting inflammation and enhancing host defenses (51–54).

Several DUB inhibitors showed marked efficacy in clearing intracellular *Salmonella* infections. These include HBX 19818, 6-Thioguanine, BAY 11-7082, BC-1471, DI-591, GK16S, OTUB1/USP8-IN-1, the USP25/USP28 inhibitor AZ-1, and USP8-IN-3. These inhibitors target key enzymes within the host’s ubiquitin-proteasome system, including USP7, USP2, USP21, STAMBP, USP14, UCHL1, OTUB1, USP8, and USP25/USP28. For instance, USP8 inhibition has been linked to autophagy modulation and enhanced *Salmonella* clearance (17). USP7 plays a role in inflammasome regulation and NF-κB stabilization, with its inhibition significantly increasing TNF-α levels in our data set, highlighting its complex role in infection (55). USP7 inhibition by three different inhibitors resulted in a significant increase in TNF-α levels following *Salmonella* infection. Other DUBs like UCH-L1 and OTUB1 influence bacterial invasion by modulating actin cytoskeleton dynamics and autophagy. For instance, UCH-L1 inhibition reduces *Listeria* and *Salmonella* uptake (56), while OTUB1 knockdown decreases *Yersinia* infections through its effects on RhoA dynamics (57). However, limitations of current inhibitors, such as LDN-57444’s transient activity, show the need for more robust alternatives (58).

USP25, which was consistently upregulated during *Salmonella* infection, and whose inhibitor AZ-1 (a dual USP25/USP28) was identified as a top hit in our compound screen (Table 1), emerged as a promising target for host-directed antimicrobial therapy. To directly assess its functional role, we performed siRNA-mediated knockdown in macrophages. Silencing USP25 alone significantly reduced intracellular *Salmonella* burden, confirming its role as a host factor that facilitates bacterial persistence. Importantly, the AZ-1 treatment did not further enhance bacterial clearance in USP25 knockdown cells, supporting the compound’s specificity for this target.

AZ-1, a USP25/USP28 inhibitor, showed broad-spectrum potential by reducing bacterial loads of *P. aeruginosa, K. pneumoniae*, and *A. baumannii*. While these pathogens are typically classified as extracellular, they are known to have intracellular phases, particularly within macrophages, demonstrating their ability to exploit intracellular niches during infection (59–61). While AZ-1 was effective against *Salmonella, P. aeruginosa, K. pneumoniae*, and *A. baumannii*, it showed limited efficacy against *F. novicida*. This may be due to *F. novicida*’s resistance to oxidative burst mechanisms (62, 63). Although we did not perform USP28 knockdown or evaluate USP25 protein levels and substrate ubiquitination, these experiments should be future directions to clarify AZ-1’s selectivity and downstream mechanisms. While AZ-1’s dual specificity complicates interpretation, no highly selective USP25 inhibitors were available at the time of this study. Future efforts will focus on developing more selective compounds to confirm USP25-specific effects. Although we did not evaluate USP25 protein levels or substrate ubiquitination following AZ-1 treatment, these remain important directions for elucidating its mechanism of action.

Interestingly, recent studies have shown that USP25 is also induced during *Mycobacterium tuberculosis* infection, where it promotes antimycobacterial responses in macrophages, as demonstrated using USP25 knockout models (64). However, the application of AZ-1 in this context has not yet been explored. Testing AZ-1 in additional infection models, including *Mycobacterium* and other chronic pathogens, will further define its therapeutic scope.

The exact mechanism by which AZ-1 exerts its therapeutic effects remains unclear. However, AZ-1 modulated NF-κB signaling by suppressing phosphorylation at early stages of infection and stabilizing unphosphorylated NF-κB at later stages. *Salmonella* infection activates several host pathways, including ERK1/2 and NF-κB, both of which support intracellular survival. ERK1/2 promotes cyclooxygenase-2 (COX-2) expression via the protein kinase A (PKA) pathway (30) while NF-κB drives inflammatory and pro-survival responses. Prior studies have shown that USP25 deficiency increases p-ERK1/2 and mitogen-activated protein kinase (MAPK) activation (65), although only minimal MAPK modulation has been observed in IL-17-stimulated macrophages (66). In our model, AZ-1 treatment stabilized TRAF3 during late-stage infection, consistent with delayed USP25-mediated regulation. While AZ-1 suppressed NF-κB phosphorylation at 8 hpi, this effect was not sustained by 26 hpi, suggesting dynamic regulation. The increased abundance of unphosphorylated NF-κB at both time points further supports a shift in signaling state. Although these observations implicate NF-κB as a downstream target of AZ-1, we acknowledge that a direct mechanistic link between USP25 inhibition and altered NF-κB signaling has not yet been established. The precise molecular intermediates—such as ubiquitination substrates or interacting partners—remain undefined. Addressing this mechanistic gap will require biochemical validation of USP25 substrates and signaling complexes in the context of infection. Additionally, we did not examine whether AZ-1 affects *Salmonella* virulence factor expression (e.g., SPI-1/SPI-2), but future studies will explore whether host-directed modulation of pathways like p53 or NF-κB may indirectly influence bacterial gene regulation.

Despite the promise of DUB inhibitors like AZ-1, the lack of specific inhibitors for many DUBs remains a challenge. Upregulated DUBs during *Salmonella* infection, such as USP11 (67), OTUD7B (68–70), and OTUD1, which are involved in innate immunity, may present future therapeutic targets. Conversely, DUBs downregulated in infection, like OTUD4 (71) and UCH-L5 (72), may indicate disruptions in host defense mechanisms that warrant further exploration.

*In vivo*, AZ-1 treatment reduced early bacterial colonization and attenuated weight loss but did not improve overall survival, indicating that monotherapy may be insufficient for advanced infection stages. The oral dose of 20 mg/kg was selected based on prior studies and tolerability (73, 74); however, further pharmacokinetic analysis and dose optimization are warranted to enhance its therapeutic potential as host-directed antimicrobial. Importantly, AZ-1 exhibited no direct antibacterial activity in axenic culture, reinforcing its mechanism as a host-targeted therapeutic. The observed reduction in intracellular *Salmonella* burden following USP25 knockdown further supports USP25 as a key target, though potential off-target effects—including contributions from USP28 or unrelated pathways—cannot yet be ruled out. These findings show the potential of AZ-1 as a host-directed agent and suggest that combining it with conventional antibiotics may offer synergistic benefits by simultaneously addressing intracellular and extracellular bacterial reservoirs. Ultimately, our results advance the case for host-targeted strategies while emphasizing the need for greater specificity and mechanistic insight to guide future therapeutic development.

In summary, this study highlights USP25 as a candidate host factor that may support *Salmonella* persistence within macrophages, based on its infection-induced upregulation and the reduced bacterial burden observed following USP25 knockdown. However, as most functional insights were derived using AZ-1—a dual USP25/USP28 inhibitor—the distinct contributions of each DUB remain to be fully elucidated. AZ-1 demonstrated robust intracellular antibacterial activity and favorable immunomodulatory effects, yet its limited efficacy as monotherapy *in vivo* underscores the need for combination strategies, particularly with antibiotics capable of targeting extracellular pathogens. Given the poor intracellular penetration of many conventional antibiotics (75), such dual approaches may offer enhanced therapeutic potential against persistent infections. More broadly, our high-throughput screening of UPS-targeting compounds revealed multiple modulators that bolster macrophage antimicrobial responses without compromising host viability. These findings bring potential promise of the ubiquitin-proteasome system—and DUBs in particular—as tractable targets for host-directed therapies aimed at overcoming antimicrobial resistance.

## MATERIALS AND METHODS

### Cell and *Salmonella* cultures

RAW 264.7 macrophages, a murine macrophage cell line, were cultured in Dulbecco’s modified Eagle medium (DMEM) supplemented with 10% fetal bovine serum (FBS) and 1% penicillin-streptomycin.

*Salmonella enterica* serovar Typhimurium strains 12023 and UK-1 (c3761) were grown overnight at 37°C in Lennox broth (LB) Miller broth with shaking, as previously described (76, 77). Overnight cultures were diluted 1:80 in fresh LB to an OD_600_ of 0.05 and grown to mid-log phase (OD_600_ ≈ 0.5), then washed with phosphate-buffered saline (PBS) and resuspended in cell culture medium. GFP-expressing *Salmonella* UK-1 was generated by electroporating the pON::sfGFP plasmid as described previously (78).

### High-throughput screening of ubiquitination pathway modulators for enhancing macrophage-mediated bacterial clearance

We conducted an HTS to identify ubiquitination pathway modulators that enhance intracellular *Salmonella* clearance in macrophages (Fig. 1). The compound library included 75 E1/E2/E3 ligase inhibitors, 59 DUB inhibitors, 58 E3 ligase ligands, 52 apoptosis modulators, and 50 proteasome-targeting compounds. Additional categories included MDM-2/p53 inhibitors (26), autophagy modulators (16), molecular glues (14), p97 inhibitors (11), and NF-κB inhibitors (9), along with compounds targeting Bcl-2, Akt, IKK, cathepsin, and other immune-related pathways.

GFP-expressing *Salmonella enterica* serovar Typhimurium was cultured overnight in LB Miller at 37°C with shaking, subcultured to OD_600_ = 0.05, and grown to mid-log phase (OD_600_ ≈ 0.5). RAW 264.7 macrophages were seeded in 96-well plates and infected at an MOI of 30:1. After infection, cells were treated with a DUB inhibitor library and incubated for 2 or 24 h. Following treatment, cells were washed with Dulbecco's phosphate-buffered saline (DPBS; containing Ca²^+^/Mg²^+^), fixed in 4% paraformaldehyde (PFA), permeabilized with 0.1% Triton X-100, and stained with Hoechst and CellMask Red to facilitate the assessment of cell viability number and bacterial load. Imaging was performed using Cytation 5 (Agilent) to capture GFP-*Salmonella*, Hoechst-stained nuclei, and cytoplasm. Bacterial burden was quantified and normalized to cell count. The efficacy of each DUB inhibitor was assessed by comparing intracellular bacterial loads in treated versus control wells using fluorescence imaging. This enabled the identification of compounds that significantly reduced bacterial burden in macrophages. Statistical analysis was performed in RStudio (v.4.3.0) using the ggplot2, dplyr, and ggrepel packages. Mean values of replicates were calculated, followed by *t*-tests to obtain *P*-values and fold changes relative to vehicle control. Significant hits were visualized in volcano plots showing log10 fold change versus –log10 *P*-value. Cell viability was assessed using 4′,6-diamidino-2-phenylindole (DAPI)-stained nuclei counts. Average counts per condition were calculated and normalized to vehicle control. Statistical significance was determined using *t*-tests, and results were visualized in volcano plots (*x*-axis: % viability, *y*-axis: –log10 *P*-value) using R packages tidyverse, ggplot2, and ggrepel.

### *Salmonella* infection studies

RAW 264.7 macrophages were infected with GFP-labeled or wild-type *Salmonella* at an MOI of 30:1. After 1 h, gentamicin was added at a concentration of 100 µg/mL for 1 h, washed with 1× DPBS, and lowered to 25 µg/mL gentamicin for the remaining amount of time. For the infections that included inhibitors, cells were infected for 1 h and washed with PBS, and then media was added that included inhibitors at indicated concentrations (in the absence of gentamicin), including AZ-1, LDN-57444, and ML323, to evaluate their impact on bacterial clearance. Appropriate vehicle controls were used at the same volumetric concentration of DMSO as the inhibitor-containing stocks.

### Gentamicin protection assay

The assay was performed as previously described with minor modifications (17). Briefly, RAW 264.7 macrophages were infected with *Salmonella* Typhimurium UK-1 grown to mid-log phase. After 1 h of infection, extracellular bacteria were removed by washing and treatment with 100 µg/mL gentamicin for 1 h. Subsequently, the medium was replaced with fresh medium containing 25 µg/mL gentamicin to prevent extracellular regrowth, and cells were treated with test compounds where indicated. Infected macrophages were incubated for either 2 or 24 h post-infection. At the specified time points, cells were washed and lysed with 0.1% Triton X-100 in PBS to release intracellular bacteria, and serial dilutions of lysates were plated on LB agar for CFU enumeration. This allowed quantification of viable intracellular bacteria to assess bacterial survival and replication within host cells.

### USP25 knockdown and subsequent infection

USP25 knockdown in RAW 264.7 macrophages was achieved via siRNA transfection. Cells were transfected with 5 pmol of USP25-specific siRNA (Invitrogen, Cat# 4390771) or a non-targeting scrambled control siRNA (Invitrogen, Cat# 4390843) using Lipofectamine RNAiMAX (Thermo Fisher Scientific), according to the manufacturer’s instructions.

Knockdown efficiency was evaluated 48 h post-transfection using quantitative reverse transcription PCR (qRT-PCR). Total RNA was extracted, reverse transcribed into cDNA using the iScript cDNA Synthesis Kit (Bio-Rad, Cat# 1708841), and amplified with USP25-specific primers (Bio-Rad, Cat# 10025636). Gene expression was normalized to glyceraldehyde 3 phosphate dehydrogenase (GAPDH), and relative expression levels were calculated using the ΔΔCt method.

To assess the impact of USP25 silencing on bacterial survival, a gentamicin protection assay was performed. At 48 h post-transfection, RAW 264.7 cells were infected with mid-log-phase *Salmonella enterica* serovar Typhimurium strain UK-1 at an MOI of 30. The gentamicin protection assay was then conducted as described previously (79).

### Intramacrophage *Francisella novicida* growth assay

Infections of murine bone marrow-derived macrophages (BEI Resources, NR-9456) were performed as previously described (80). Briefly, 4.5 × 10⁴ cells were seeded without antibiotics, washed, and infected at an MOI of 0.1. Plates were centrifuged at 1,000 × *g* for 30 min at room temperature and incubated at 37°C for an additional 30 min. Cells were then treated with 50 µg/mL gentamicin for 30 min to eliminate extracellular bacteria, followed by washing. Infected cells were treated with the indicated drug concentrations and lysed either immediately or at 18 hpi using 0.1% Triton X-100. Lysates were diluted in tryptic soy broth supplemented with 0.1% cysteine and plated for CFU enumeration. Experiments were conducted with at least four technical and three biological replicates.

### Evaluation of AZ-1 on intramacrophage growth of ESKAPE pathogens

RAW 264.7 cells were maintained in Dulbecco’s modified Eagle’s medium (GenClone 25-500) supplemented with 10% FBS and 1% Pen Strep (GenClone, 25-512) in a 37°C humidified incubator with 5% CO_2_. Two days prior to infection, cells were scraped, washed, and seeded in 96-well plates at a density of 5 × 10^4^ cells/well in 100 µL of DMEM supplemented with 10% FBS without antibiotics. The day before infection, *P. aeruginosa* PAO1*, K. pneumoniae* KPPR1*,* and *A. baumannii* MAB103 were grown in LB in a 37°C incubator shaking at 220 revolutions per minute (rpm). On the day of infection, eukaryotic cells were detached with trypsin at 37°C for 10 min and counted. Bacteria were washed and resuspended in PBS, then added to macrophages at an MOI of 10. Infection was facilitated by centrifugation (500 × *g*, 30 min), followed by a 30 min incubation at 37°C. Post-infection, cells were washed and treated with 100 µg/mL gentamicin for 1 h to eliminate extracellular bacteria. Medium was then replaced with 25 µg/mL gentamicin-containing DMEM with either 10 µM AZ-1 or vehicle (DMSO) and incubated for 4 h. Cells were lysed with 0.05% Triton X-100, and lysates were plated on LB agar to determine intracellular bacterial CFUs.

### qPCR analysis

To assess DUB expression during *Salmonella* infection, we performed qRT-PCR using a custom primer array covering all characterized murine DUBs (Bio-Rad). RAW 264.7 macrophages were infected with *Salmonella* Typhimurium (MOI 30:1) as described above. Total RNA was extracted from infected cells and reverse transcribed into cDNA using iScript Supermix (Bio-Rad). qPCR was performed on a C1000 Touch Thermal Cycler and analyzed with CFX Maestro Software (Bio-Rad). Relative transcript levels were calculated using the comparative Ct method (ΔΔCt), normalized to housekeeping genes (GAPDH, Rpl27, Hprt, and β-actin), as previously described (81, 82).

### Growth curve analysis

Overnight cultures of *Salmonella* Typhimurium UK-1 were diluted to OD_600_ = 0.05 in LB Miller and incubated in a 96-well plate (200 µL/well) containing test compounds (10 µM), DMSO vehicle control, ampicillin (100 µg/mL, positive control), and media-only blanks. Bacterial growth was monitored at 37°C with continuous shaking using a Cytation 3 plate reader (Agilent), with OD_600_ readings taken every 30 min for 24 h. Data were analyzed in R (v.4.3.0) and visualized using standard plotting packages (ggplot2, dplyr, tidyr). Growth curves were normalized to vehicle controls and plotted with standard deviations.

### TNF-α ELISA

Following infection with wild-type *Salmonella* (as described above), cell culture supernatants were collected at 24 hpi and analyzed using a TNF-α ELISA kit (Invitrogen). TNF-α concentrations were calculated from a standard curve and normalized to vehicle controls. Statistical significance was assessed by pairwise *t*-tests with Benjamini-Hochberg correction for multiple comparisons. Data visualization and significance annotation were performed using R (ggplot2) and GraphPad Prism.

### Western blot analysis

Following the infections and treatments, cells were washed with PBS, lysed in radioimmunoprecipitation assay buffer (RIPA) buffer, and incubated on ice with intermittent vortexing. Lysates were sonicated and cleared by centrifugation. Protein concentrations were determined using a bicinchoninic acid assay (BCA) assay (Thermo Scientific). Equal amounts of protein were separated by SDS-PAGE (4–12% gradient gels), transferred to polyvinylidene fluoride (PVDF) membranes, and probed with primary antibodies against NF-κB (Cell Signaling, 8242T), p-NF-κB (Cell Signaling, 3033S), ERK1/2 (Cell Signaling, 4695T), p-ERK1/2 (Cell Signaling, 4370T), TRAF3 (Cell Signaling, 4729T), USP25 (Santa Cruz, sc-398414), and β-actin (Santa Cruz, sc-47778). Detection was performed using appropriate horseradish peroxidase (HRP)-conjugated secondary antibodies: goat anti-rabbit IgG (Invitrogen, 31460), goat anti-mouse IgG (Invitrogen, 31430), and mouse IgG1 BP-HRP (Santa Cruz, sc-525408) for USP25. Bands were visualized by enhanced chemiluminescence.

### QUANTI-Blue assay with RAW 264.7-Blue cells

RAW-Blue cells (InvivoGen), which express an NF-κB/AP-1-driven SEAP reporter, were used for gentamicin protection assays as described above. At 4, 8, and 26 h post-infection—or prior to infection (baseline)—20 µL of supernatant was mixed with 180 µL of QUANTI-Blue solution (InvivoGen) and incubated at 37°C. Absorbance was measured at 620/655 nm using a Cytation3 plate reader. For LPS stimulation studies, cells were treated with vehicle or AZ-1, with or without 500 ng/mL LPS (Millipore Sigma, L6143). Supernatants were collected at the same time points, mixed with QUANTI-Blue solution, incubated for 16 h at 37°C, and analyzed for SEAP activity as described above.

### Cytotoxicity and viability assay

The cytotoxicity and viability of RAW 264.7 cells treated with bortezomib (10 µM), AZ-1 (10 µM), or DMSO (vehicle control) after 24 h and digitonin (positive control for complete lysis) after 15 min were evaluated using the Cytation 3 (Agilent) multifunctional reader. The MultiTox-Fluor Multiplex Cytotoxicity Assay was employed to determine the ratio of cytotoxicity to viable cells. Data are presented as mean ± SEM from three independent experiments and were analyzed using a one-way ANOVA followed by Tukey’s *post hoc* test for multiple comparisons.

### Murine infection

*Salmonella enterica* serovar Typhimurium 12023 (nalidixic acid-resistant) was grown overnight in LB Lennox with 30 µg/mL nalidixic acid at 37°C with shaking (220 rpm). A subculture was prepared the next day, grown to mid-log phase, washed three times in PBS, and resuspended to achieve the desired CFU for infection. AZ-1 was prepared at 100 mM in DMSO. For treatment, 7.1 µL of stock was diluted in 100 µL of sterile PBS to deliver 20 mg/kg via oral gavage. The vehicle control consisted of 7.1 µL DMSO in 100 µL PBS. Eight-week-old female C57BL/6J mice (Jackson Labs, strain 000664) were housed under specific pathogen-free conditions in the University of Florida’s Department of Veterinary Medicine facility, with a 12 h light/dark cycle and access to standard chow and water. Mice were randomly assigned to treatment groups and identified using ear punches. Baseline body weights were recorded prior to treatment. To disrupt resident gut microbiota, mice were gavaged with 100 µL of freshly prepared 200 mg/mL streptomycin sulfate in sterile water 24 h prior to infection. Ten minutes before bacterial administration, mice were given 50 µL of 0.3 M sodium bicarbonate via oral gavage to neutralize gastric acid. Infection was performed via oral gavage with 1 × 10⁸ CFU of *Salmonella* in 50 µL PBS. AZ-1 or vehicle was administered daily by oral gavage beginning 12–16 h after infection and continued throughout the study. To support survival during acute infection, mice were provided soft chow starting on day 2 post-infection. Mice were monitored daily for weight loss and clinical symptoms. Humane endpoints were defined as death or ≥20% loss of baseline body weight. On day 2 post-infection, fecal pellets were collected to assess bacterial burden. Pre-weighed 1.5 mL microcentrifuge tubes were used to determine fecal sample weight. Mice were placed individually in sterile pup cups until fresh droppings were produced, which were collected using sterile pipette tips. Each fecal sample was resuspended in 1 mL of sterile PBS and vortexed at high speed for 15 min to create a uniform slurry. Serial dilutions (30 µL in 270 µL PBS) were prepared, and 100 µL of 10⁻¹, 10⁻⁴, and 10⁻⁵ dilutions were plated in triplicate onto LB Lennox agar supplemented with 30 µg/mL nalidixic acid. Plates were incubated overnight at 37°C, and CFUs were counted to determine bacterial load (CFU/g feces).

## Data Availability

The authors certify that they will comply with ASM's Data Policy. Data supporting the findings of this study will be made publicly available upon request.

## SUPPLEMENTAL MATERIAL

Supplemental material mbio.00312-25-s0001.pdf Supplemental figures and table.

ASM does not own the copyrights to Supplemental Material that may be linked to, or accessed through, an article. The authors have granted ASM a non-exclusive, world-wide license to publish the Supplemental Material files. Please contact the corresponding author directly for reuse.
