# Supplementary material for: Targeting deubiquitinating enzymes and ubiquitin pathway modulators to enhance host defense against bacterial infections
Source: mBio. 2025 Sep 3;16(10):e00312-25. doi: 10.1128/mbio.00312-25 (PMC12506015; doi:10.1128/mbio.00312-25)
Supplement: Supplemental material — Supplemental figures and table. [file mbio.00312-25-s0001.pdf]

Supplementary data

Supplementary figures:

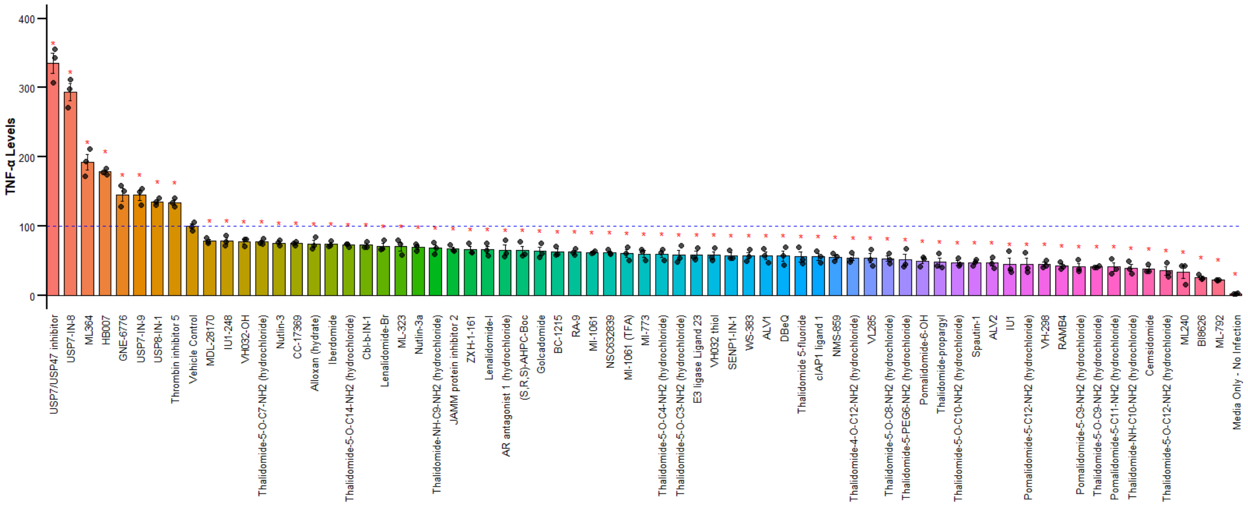

Figure S1. Evaluation of select DUB inhibitors in promoting increased or decreased TNF-α secretion from cells infected with *Salmonella*. RAW264.7 cells were infected with GFP-labeled *Salmonella* (MOI 30:1) for 24 hours as in ***Figure 1***. After this period, cell culture supernatants were collected and evaluated for TNF-α presence using ELISA. For each plate, the average TNF-α level from vehicle control-infected cells was set as 100% to calculate the effect change. The data analysis was conducted using R. TNF-α data were loaded, and p-values and fold changes compared to the vehicle control were calculated using t-tests, followed by the Benjamini-Hochberg correction for multiple testing. Compounds with adjusted p-values < 0.05 were considered significant. Only the significant compounds that did not lower cell viability as well as controls are shown.

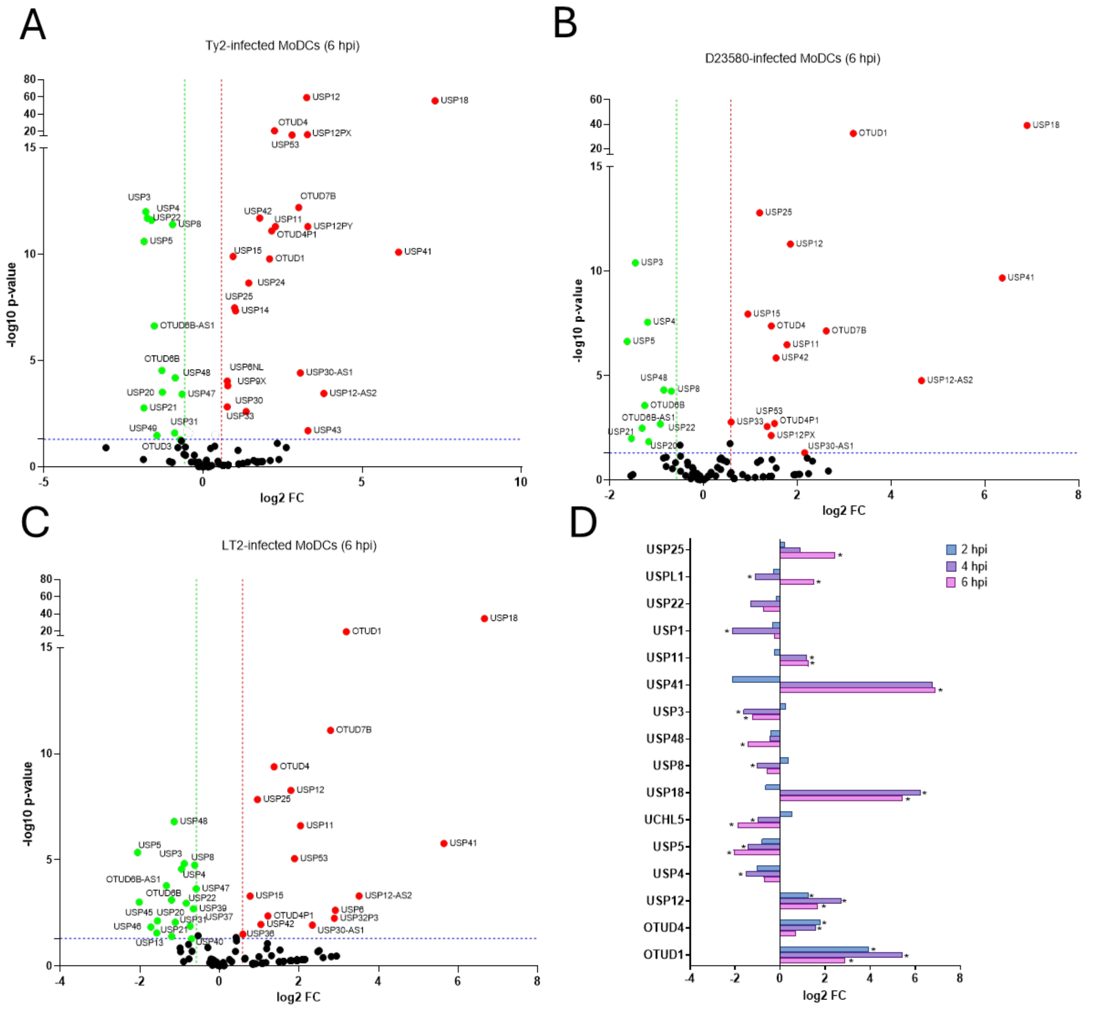

Figure S2. The expression of deubiquitinating enzymes is regulated during infection of human moDCs with various *Salmonella* strains. (A-C). Differential expressions of human DUB transcripts in moDCs infected with Ty2, D23580, and LT2 strains of *Salmonella* at 6 hpi. The figure illustrates the RNA sequencing results depicting the expression levels of DUBs in monocyte-derived dendritic cells (moDCs) infected with three distinct *Salmonella* strains: Ty2 (serovar Typhi strain) **(A)**, D23580 (*Salmonella* serovar Typhimurium clinical isolate, iNTS strain) **(B)**, and LT2 (serovar Typhimurium, laboratory strain) **(C),** at 6 hours post-infection (hpi). The data were obtained through dual RNA sequencing of *Salmonella*-infected moDCs, where infected cells were sorted using fluorescence-activated cell sorting (FACS) at 6 hpi. **(D.)** Differential expression of DUBs in MoDCs infected with *Salmonella* LT2 strain over the time course of 2, 4 and 6 hours. Log2-transformed normalized expression levels of a subset of DUBs in human MoDCs following exposure to *Salmonella* Typhimurium LT2. Cells were challenged at varying time points (2-, 4-, and 6-hours post-infection) and processed using the Smart-seq2 protocol.

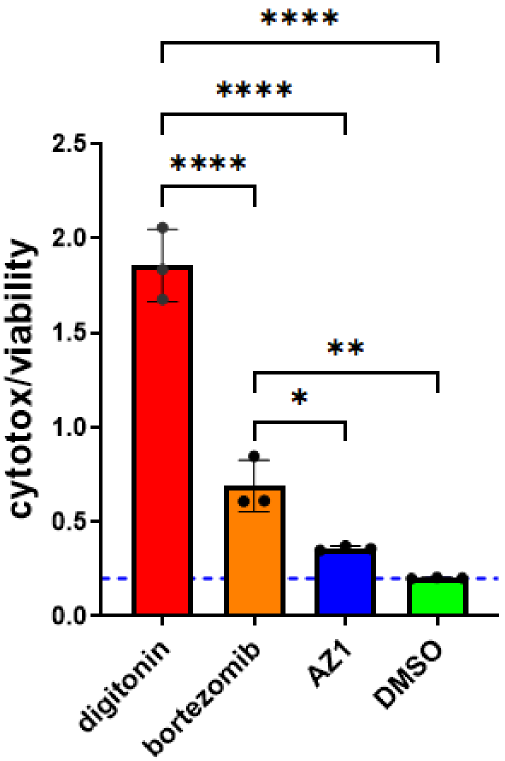

Figure S3. Assessment of Cytotoxicity/Viability in RAW 264.7 cells treated with Bortezomib and AZ-1. RAW 264.7 cells were treated with digitonin (positive control – total lysis), bortezomib (10 μM), AZ-1 (10 μM), or DMSO (vehicle control), and both cytotoxicity and viability were assessed after 24 hours of treatment. The y-axis represents the ratio of cytotoxicity to viability. Data are presented as mean ± SEM from three independent experiments. Statistical significance was determined using one-way ANOVA followed by Tukey's post-hoc test. Significant differences are indicated as follows: *$p < 0.05$, **$p < 0.01$, ***$p < 0.001$, ****$p < 0.0001$.

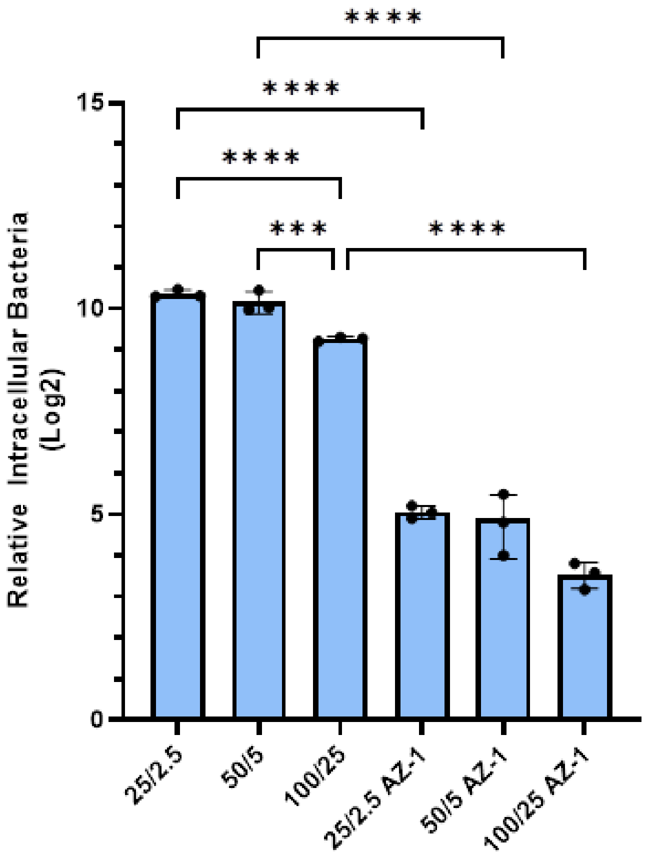

Figure S4. Impact of varying gentamicin regimens on AZ-1's efficacy in reducing intracellular *Salmonella* load. Gentamicin (25, 50, or 100 μg/mL) was applied for 1 hour post-infection of RAW 264.7 cells with *Salmonella* (MOI 30:1) to eliminate extracellular bacteria, followed by its removal. Subsequent treatments included a lower concentration of gentamicin (25, 5, or 2.5 μg/mL) combined with either the vehicle control or AZ-1. Hoechst staining (blue) labeled nuclei, GFP fluorescence (green) indicated intracellular *Salmonella*, and CellMask staining (red) highlighted cell structures, enabling visualization of both bacteria and host cells. The key for the figure is as follows: 25/2.5 represents gentamicin at 25 μg/mL for 1 hour followed by 2.5 μg/mL overnight; 50/5 represents gentamicin at 50 μg/mL for 1 hour followed by 5 μg/mL overnight; 100/25 represents gentamicin at 100 μg/mL for 1 hour followed by 25 μg/mL overnight. The regimens combined with AZ-1 are labeled as follows: 25/2.5 + AZ-1 corresponds to the 25/2.5 regimen with AZ-1 treatment overnight; 50/5 + AZ-1 corresponds to the 50/5 regimen with AZ-1 treatment overnight; and 100/25 + AZ-1 corresponds to the 100/25 regimen with AZ-1 treatment overnight.

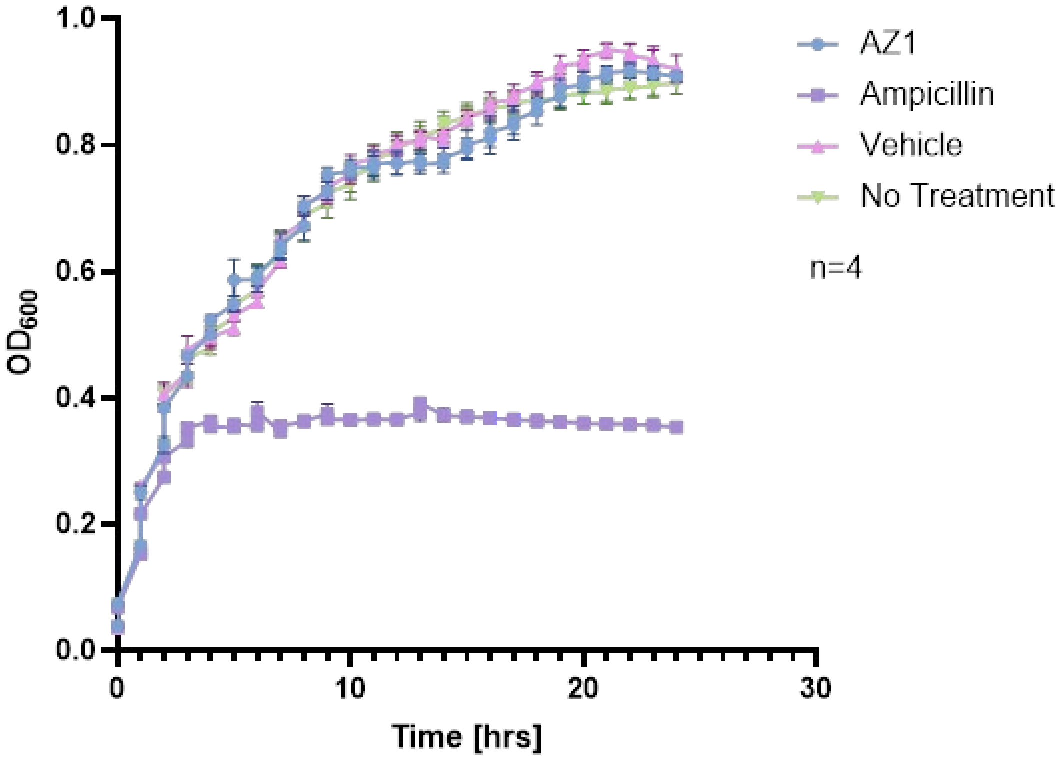

Figure S5. Growth of *Salmonella* in axenic culture with AZ-1. *Salmonella enterica* serovar Typhimurium was cultured in LB broth and treated with AZ-1, Ampicillin, Vehicle (DMSO solution), or left untreated. Optical density at 600 nm (OD_{600}) was measured over 24 hours. *n* = 4; error bars represent SEM.

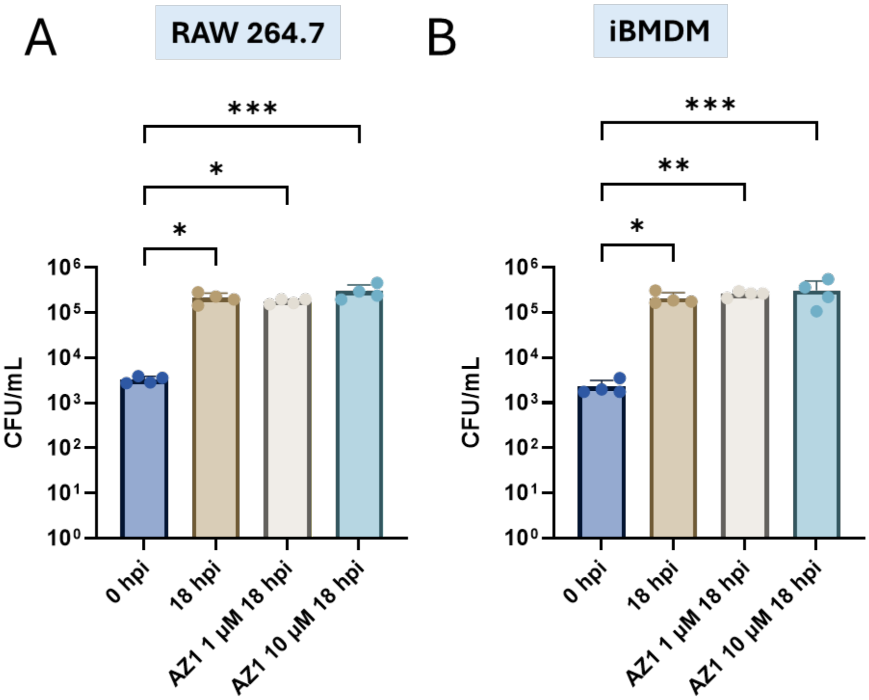

Figure S6. AZ-1 fails to support clearance of *Francisella novicida* from infected macrophages. Immortalized murine bone marrow-derived macrophages were infected with *Francisella novicida* at a multiplicity of infection of 0.1. After washing and gentamicin treatment to remove extracellular bacteria, the infected macrophages were treated with the indicated concentration of AZ1. The cells were lysed immediately (0 h) and 18 hours post-infection (hpi) to determine the number of colony-forming units (CFUs). Data from RAW 264.7 cells **(A)** and iBMDMs **(B)** are shown. Bars represent the mean CFU ± standard deviation (SD). Statistical significance was assessed using ordinary one-way ANOVA followed by Tukey's post hoc test.

Supplementary tables:

Table S1. Related to Figure 3. Effects of ubiquitin pathway-modulating compounds on TNF-α expression relative to a vehicle control consisting of DMSO.

Compound	*Mean TNF-α value*	*Difference from Vehicle Control*	*-log10 p-value*
(S,R,S)-AHPC-Boc	65.1380398	-34.86196021	1.813070121
ALV1	57.58888764	-42.41111236	2.216248776
ALV2	47.31357104	-52.68642896	3.017366787
AR antagonist 1 (hydrochloride)	65.73492542	-34.26507458	1.584361413
Alloxan (hydrate)	74.95751502	-25.04248499	1.70270195
Ampicillin	35.73607518	-64.26392482	2.207699043
BC-1215	63.33117764	-36.66882236	2.614724865
BI8626	26.45984993	-73.54015008	3.776131117
CB-5083	45.53600381	-54.46399619	2.967210502
CB-5339	3.190529727	-96.80947028	2.880649361
CC-17369	75.28486456	-24.71513544	2.074660138
Cbl-b-IN-1	72.82177338	-27.17822662	2.335847689
Cemsidomide	38.60972009	-61.39027992	3.641863907
DBeQ	57.12053673	-42.87946327	1.824272371
DUB-IN-1	4.460161697	-95.53983831	3.250693211
DUB-IN-3	1.30632988	-98.69367012	2.896289425
E3 ligase Ligand 23	58.76081652	-41.23918348	2.420190106
EOAI3402143	34.36705116	-65.63294884	2.966586631
GNE-6776	145.8538454	45.85384543	1.600498574
Golcadomide	63.92194542	-36.07805459	1.986540035
HB007	178.9136463	78.91364626	4.050118326
Hinokiflavone	22.73778038	-77.26221962	3.75155706
IU1	45.46134032	-54.53865968	1.761290042
IU1-248	78.73024179	-21.26975822	1.685636248
Iberdomide	74.23003543	-25.76996457	2.237433212
JAMM protein inhibitor 2	67.47174757	-32.52825243	2.625443537
Lenalidomide-Br	71.50311247	-28.49688754	2.178212481
Lenalidomide-I	66.25954861	-33.74045139	2.065137871
MDL-28170	78.93570953	-21.06429047	1.96716469
MG-115	27.98284781	-72.01715219	2.872331084
MI-1061	62.56512042	-37.43487958	2.216563692
MI-1061 (TFA)	60.87184378	-39.12815623	2.156863269
MI-773	60.28877963	-39.71122037	2.519015472
ML-323	70.67955759	-29.32044241	1.578740342
ML-792	22.42622459	-77.57377542	2.709849049
ML240	33.81688845	-66.18311155	2.025931525
ML364	192.5855559	92.58555586	2.035616559
Media Only - No Infection	2.645411116	-97.35458889	2.968189453
N-Ethylmaleimide	54.00252199	-45.99747801	1.817026466

NMS-859	55.71677097	-44.28322903	3.06525189
NSC632839	62.47437923	-37.52562077	2.722509091
NSC697923	65.25492576	-34.74507425	2.671428801
Nutlin-3	76.09308595	-23.90691405	2.134630683
Nutlin-3a	69.79487441	-30.20512559	2.486997548
Oprozomib	4.82647276	-95.17352724	3.921556144
Pomalidomide-5-C11-NH2 (hydrochloride)	41.3391341	-58.6608659	2.529008986
Pomalidomide-5-C12-NH2 (hydrochloride)	45.20237659	-54.79762342	1.875251606
Pomalidomide-5-C9-NH2 (hydrochloride)	41.84211881	-58.15788119	2.952835414
Pomalidomide-6-OH	50.24583612	-49.75416388	2.980575943
RA-9	62.84262206	-37.15737794	2.816769268
RA190	19.35713175	-80.64286825	2.835476949
RA375	14.75501944	-85.24498056	4.192652669
RAMB4	42.70269873	-57.29730128	3.543126351
SENP1-IN-1	58.09022428	-41.90977572	2.886654502
Siremadlin	9.558657207	-90.4413428	3.397948782
Spautin-1	47.43606865	-52.56393136	3.363437752
Thalidomide 5-fluoride	56.23919428	-43.76080573	1.89462841
Thalidomide-4-O-C12-NH2 (hydrochloride)	54.39712175	-45.60287825	2.962261576
Thalidomide-5-O-C10-NH2 (hydrochloride)	47.79784841	-52.20215159	3.345621902
